# The impact of serological testing implementation on tick-borne encephalitis detection in Poland

**DOI:** 10.1371/journal.pone.0323022

**Published:** 2025-05-16

**Authors:** Joanna Zajkowska, Elżbieta Waluk, Renata Świerzbińska, Justyna Dunaj, Olga Zajkowska, Dominik Wawrzuta, Jolanta Niścigorska-Olsen, Marek Matukiewicz, Barbara Oczko-Grzesik, Daniel Veltze, Katarzyna Bernacka-Andrzejewska, Katarzyna Burchart-Adamczyk, Ewa Dutkiewicz, Jadwiga Maciukajć, Krystyna Konieczny, Danuta Malcher-Bober, Dorota Dybowska, Małgorzata Hapyn-Rocha, Monika Marsik-Styrkosz, Grzegorz Kmak, Monika Bociąga-Jasik, Magdalena Byś-Chrzanowska, Iwona Paradowska-Stankiewicz

**Affiliations:** 1 Department of Infectious Diseases and Neuroinfections, Medical University in Białystok, Białystok, Poland; 2 Faculty of Economic Sciences, University of Warsaw, Warsaw, Poland; 3 Department of Radiotherapy, Maria Sklodowska-Curie National Research Institute of Oncology, Warsaw, Poland; 4 Department of Infectious, Tropical Diseases and Immune Deficiency, Pomeranian Medical University, Szczecin, Poland; 5 Infectious Diseases Department, University Hospital of Karol Marcinkowski in Zielona Góra, Zielona Góra, Poland; 6 Department of Infectious Diseases and Hepatology, Medical University of Silesia, Bytom, Poland; 7 Provincial Sanitary and Epidemiological Station in Gdańsk, Gdańsk, Poland; 8 Department of Infectious Diseases, Ludwik Perzyna Complex Hospital, Kalisz, Poland; 9 Infectious Diseases Department, Healthcare Team Hospital in Busko-Zdrój, Busko-Zdrój, Poland; 10 Observation and Infectious Diseases and Viral Hepatitis Department, Specialist Hospital in Jasło, Jasło, Poland; 11 Observation and Infectious Diseases Department, Provincial Hospital in Przemyśl, Przemyśl, Poland; 12 Department of Infectious Diseases and Hepatology, Faculty of Medicine, Nicolaus Copernicus University, Bydgoszcz, Poland; 13 Observation and Infectious Diseases Department, Ludwik Rydygier Voivodship Polyclinical Hospital, Toruń, Poland; 14 Pediatric and Pediatric Neurology Department, The John Paul II Specialist Hospital in Kraków, Kraków, Poland; 15 Department of Infectious and Tropical Diseases, Jagiellonian University Medical College, Kraków, Poland; 16 Clinical Infectious Diseases Department, Medical Center in Łańcut, Łańcut, Poland; 17 Department of Epidemiology, National Institute of Public Health-National Institute of Hygiene in Warsaw, Warsaw, Poland; Texas A&M University, UNITED STATES OF AMERICA

## Abstract

**Introduction:**

Tick-borne encephalitis virus (TBEV) infections remain underreported in Poland, leading to inadequate public awareness of the potential severity of the disease and its preventive measures.

**Aim:**

This study aims to assess the incidence of tick-borne encephalitis (TBE) in non-endemic regions of Poland by analyzing serum or cerebrospinal fluid (CSF) samples from patients with neuroinfections of unknown origin.

**Materials and Methods:**

In this study, 29 departments specializing in neuroinfections were involved. Hospitals were chosen from regions classified as non-endemic with limited TBE reporting. Patients treated between April 1, and December 31, who had neuroinfections of unknown origin, were enrolled. Their CSF or serum samples underwent serological diagnosis of TBE using the Virotech ELISA kit at the Immunoserology Laboratory of the Medical University of Białystok. In addition, we used questionnaires to collect clinical and epidemiological data from patients,

**Results:**

Among 766 patients, 124 exhibited serum or CSF antibodies against TBEV. None of them were vaccinated against TBE. The highest positivity rates were observed in Małopolskie (31%), Świętokrzyskie (24%), and Dolnośląskie (22%) voivodeships. In contrast, the Kujawsko-Pomorskie (2%), Pomorskie (3%), and Zachodniopomorskie (7.5%) voivodeships showed the lowest ratios. All patients reported their place of residence as a potential source of infection, some also mentioning outdoor activities, travel, work, and unpasteurized dairy consumption.

**Conclusions:**

This study reveals a notable incidence of TBE infections in patients with nonspecific neuroinfections residing in regions historically seen as non-endemic. These findings emphasize the need for improved reporting and educational initiatives to raise awareness of the risk of TBE.

## Introduction

### Background

Tick-borne encephalitis (TBE) is an infectious disease caused by a TBE virus (TBEV) that belongs to the *Flaviviridae* family, a group of viruses recognized for their ability to trigger severe neurological symptoms [1]. This disease is transmitted primarily by ticks, but it is possible to acquire infection by consuming infected unpasteurized dairy products [2]. Although some cases of TBE may remain asymptomatic, a significant proportion, ranging from 35% to 58%, can lead to the development of long-term neurological sequelae, with a mortality of European TBEV lower than 1% [3]. The most important neurological symptoms of TBE are altered consciousness, ataxia, paresis, and tremors [4].

Given the absence of targeted antiviral therapies for TBE, resulting in only symptomatic treatment for affected patients, the testing for this etiology remains restricted in cases of viral encephalitis. Antiviral medications are applied exclusively to HSV-1 or VZV infections [5]. Preventive measures, particularly vaccination, play a key role in curbing the impact of TBE. Vaccination has proven highly effective, offering protection by conferring an effectiveness of over 95% [6]. Paradoxically, despite the existence of effective vaccines, uptake among the population remains disappointingly low. From 2011 to 2020, only 419,408 people in Poland (1.1% of the population) received TBE vaccination [7]. This predicament stems primarily from a limited public grasp of the disease’s risks. Moreover, the financial burden of vaccination, only borne by patients, exacerbates the hesitancy towards adopting preventive measures.

In Europe, TBE cases are more frequently reported among men, particularly in the 45–64 age group [8]. This pattern is likely explained by increased exposure to forested environments during work or recreational activities, as spending more than 10 hours per week in forests within endemic regions is a recognized risk factor for TBE infection [9]. In Poland, TBE reigns as the most menacing tick-borne disease [10]. Currently, two regions in Poland—Podlaskie and Warmińsko-Mazurskie voivodeships—are recognized as endemic for TBE. These regions are home to over 2.5 million people, with approximately 30% of the land area covered by forests. As popular tourist destinations, these areas attract around 1.5 million visitors annually, further increasing potential exposure to ticks. However, a theoretical analysis conducted by Stefanoff et al. [11] suggested that the entire territory of Poland could be considered endemic for TBE. To date, these theoretical findings have not been empirically confirmed.

Collectively, these challenges contribute to the underreporting of TBE cases. Many TBE diagnoses are classified under the generic code A87 of the International Classification of Diseases (ICD), which denotes viral encephalitis without a specified viral origin. This underreporting predicament carries a dual impact: first, it creates the perception of the rarity of TBE, thereby miscalculating the disease’s prevalence; second, it hampers the identification of high-risk regions warranting concentrated preventive efforts. Prior investigations have revealed that only about half of all TBE cases in Poland are accurately classified under the specific ICD code A84 [12,13].

### Aim of the study

This study aims to evaluate the occurrence and distribution of TBE in traditionally non-endemic regions of Poland by conducting systematic serological analyses of serum and cerebrospinal fluid samples obtained from patients presenting with unexplained neuroinfections. The ultimate goals are to identify emerging TBE hotspots and assess potential underreporting by comparing the findings with official statistics from the Polish National Institute of Public Health’s mandatory surveillance system.

## Methods

### Included centers

We invited all departments treating neuroinfections from regions identified as potentially having a higher-than-reported TBE incidence to participate in the study. This selection was based on previous theoretical modeling studies by Stefanoff et al. [11], which identified potential high-risk areas (according to WHO definition, ≥ 5 cases/100 000 population/year) through analysis of environmental factors conducive to TBEV transmission. Ultimately, 29 departments agreed to take part, including infectious disease units and neurology departments specializing in diagnosing and treating neuroinfections. These centers were strategically located in areas with limited official reporting of TBE cases, but preliminary data indicated the potential presence of undiagnosed TBE cases. Furthermore, these departments had the capacity to perform the serological tests required for this study. The Department of Infectious Diseases and Neuroinfections at the Medical University of Białystok oversaw and coordinated the participation of all included departments.

### Patient inclusion criteria

All patients treated within participating departments from April 1, 2018, to December 31, 2022, were considered for inclusion if they exhibited clinical indicators suggestive of TBE, such as inflammation of the central nervous system (manifesting as meningitis, meningoencephalitis, encephalomyelitis or encephaloradiculitis) but the specific type of neuroinfection was not confirmed. Initially, these patients were classified within epidemiological surveillance as cases of unspecified viral meningitis (coded A87, B00.3, B02.1), unspecified meningitis (coded G03), other viral-specific encephalitis (coded A83, A85, B00.4, B02.0, B25.8), or unspecified encephalitis (coded A86, G04.8, G04.9).

### Patient exclusion criteria

Patients who had received a flavivirus vaccination or did not provide their consent to participate were excluded from the study.

### Laboratory analysis

Serological tests were performed on serum or cerebrospinal fluid (CSF) samples. Patients with clinical symptoms of neuroinfection and a positive result for either IgM or IgG in serum, or IgM or IgG in CSF were diagnosed as TBE patients. To identify anti-TBE immunoglobulins, we used the enzyme-linked immunosorbent assay (ELISA) method, using Virotech ELISA kits (Germany). All analyses were performed at the Immunoserology Laboratory of the Medical University of Białystok. At the same time, patients were required to complete a questionnaire that captured clinical and epidemiological data.

### Ethical considerations

The study obtained approval from the Bioethics Committee of the Medical University of Białystok under Resolution R-I-002/418/2017.

All participants provided written informed consent to participate in the study, and no minors were involved; the consent process was approved by the ethics committee as part of the study protocol.

## Results

### Patient characteristics

**Fig 1:**
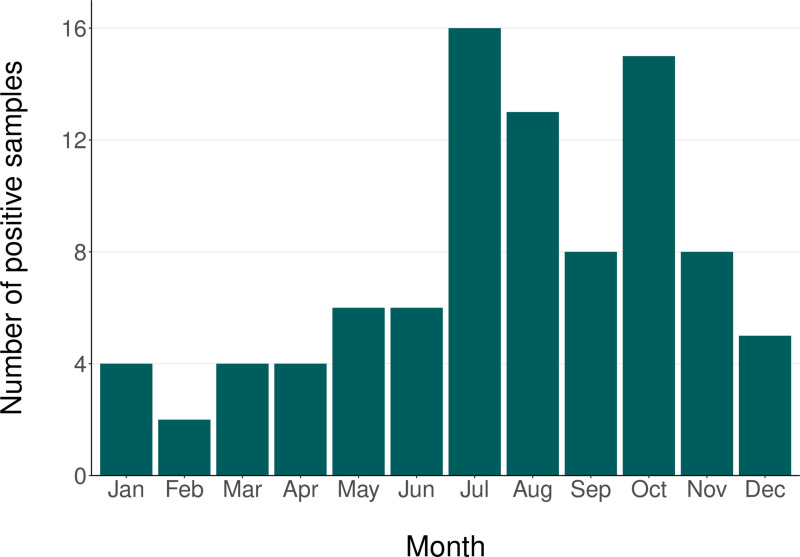
Monthly Distribution of Positive Samples.

Throughout the study period, from April 1, 2018, to December 31, 2022, we included 766 patients in our analysis, of whom 124 were diagnosed with TBE. None of them were vaccinated against TBE. The distribution of patients and confirmed TBE cases is described in Table 1, highlighting the annual count of patients, the corresponding number of confirmed cases, and the distribution of cases based on the availability of cerebrospinal fluid (CSF), serum samples, or both. In 2020, a noticeable decline in patient inclusion was observed, attributed to the significant impact of the COVID-19 pandemic on participating departments. Many of these departments, specializing primarily in infectious diseases, were transformed into specialized COVID-19 wards. Additionally, Figure 1 illustrates that the peak occurrence of new TBE infections was observed from July to October, although no months were without new TBE cases.

**Table 1:** The number of included patients and confirmed cases.

Year	Included patients	Confirmed cases	A sum of analyzed samples	Patients with only serum samples	Patients with only CSF samples	Patients with both CSF and serum samples
2018	255	33	333	93	82	79
2019	242	33	323	114	45	81
2020	73	20	93	43	8	21
2021	89	23	125	49	4	36
2022	107	15	140	65	7	34
Sum	**766**	**124**	**1014**	**364**	**146**	**251**

The average age at the diagnosis was 40 years, with a standard deviation of 19 years and a range of 3–80 years. Initial clinical diagnoses included lymphocytic, viral, or unspecified meningitis in 82 patients, neuroinfection in 1 patient, acute neuropathy in 1 patient, and febrile state in 1 patient. In 80 patients, we obtained ICD diagnoses from records that predated serological tests, as presented in Table 2.

**Table 2:** Initial diagnosis of ICD of patients diagnosed with TBE.

ICD diagnosis	Number of patients	Percentage share
A83	7	8.75%
A88.8	1	1.25%
A86	6	7.5%
A87	48	60%
G03	1	1.25%
A87.9	1	1.25%
G03	14	17.5%
G04	2	2.5%
Sum	**80**	**100%**

### Serological and Cerebrospinal Fluid Analyses

Serum antibody analyses revealed the presence of IgM antibodies in 83 samples and IgG antibodies in 49 samples. In CSF samples, IgM antibodies were detected in 47 cases, while IgG antibodies were identified in 33 cases. Furthermore, CSF parameters were analyzed in a subset of patients, unveiling a mean cell count of 197.6 per mm^3^ (standard deviation: 345.4 per mm^3^, min: 0 per mm^3^, max: 2015 per mm^3^), mean protein level of 94.9 mg/dL (standard deviation: 117.6 mg/dL, min: 18 mg/dL, max: 932 mg/dL), mean glucose level of 60.6 mg/dL (standard deviation: 14.7 mg/dL, min: 16 mg/dL, max: 123 mg/dL), and mean chloride level of 123.2 mmol/L (standard deviation: 4.9 mmol/L, min: 109 mmol/L, max: 135 mmol/L).

### Clinical characteristics

None of the diagnosed patients had received vaccinations against TBE, yellow fever, or Japanese encephalitis. In terms of additional medical conditions, nine patients reported a history of Lyme disease, while two individuals had hypertension, two had rheumatoid arthritis, and single people had diabetes mellitus, autoimmune disease, depression, CMV and herpes infection, mycoplasma infection, or undergone liver transplantation.

All patients indicated that their place of residence could have been a possible source of infection. Furthermore, 12 patients reported outdoor activities such as hunting, fishing, running, mountain biking, and mushroom picking, while 11 mentioned their workplaces as potential exposure sites. Additionally, 11 patients were suspected of contracting the infection during travel (3 of them abroad – Hungary, Corfu or Croatia), and an additional five patients reported consuming unpasteurized dairy products. A comprehensive breakdown of these potential infection sources is provided in Table 3.

**Table 3:** Possible sources of infection.

Number of reports	The possible source of infection
92	Place of residence
12	Outdoor activities – including hunting, fishing, running, mountain biking, and mushroom picking
11	Travel – destinations included Hungary, Corfu, Croatia, Lubelskie and Podlaskie Voivodeship, Gdańsk, Ustronie, Busko Zdrój, Jastrzębie Zdrój.
11	Work – two people worked in forest-related occupations.
5	Consumption of unpasteurized dairy products or cheeses.
2	Denial of the possibility of tick bites.

### Spatial distribution

**Fig 2:**
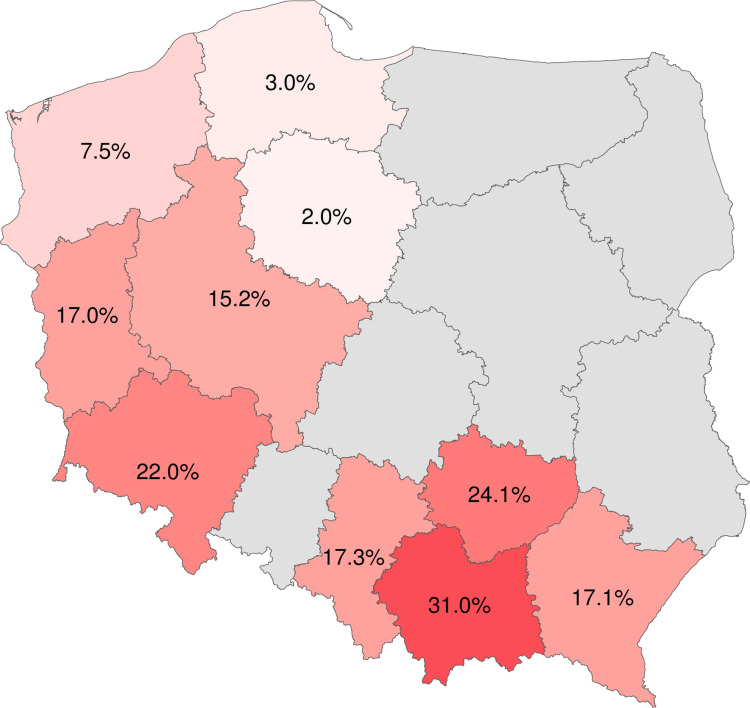
Percentage of Positive Samples by Voivodeship.

**Fig 3:**
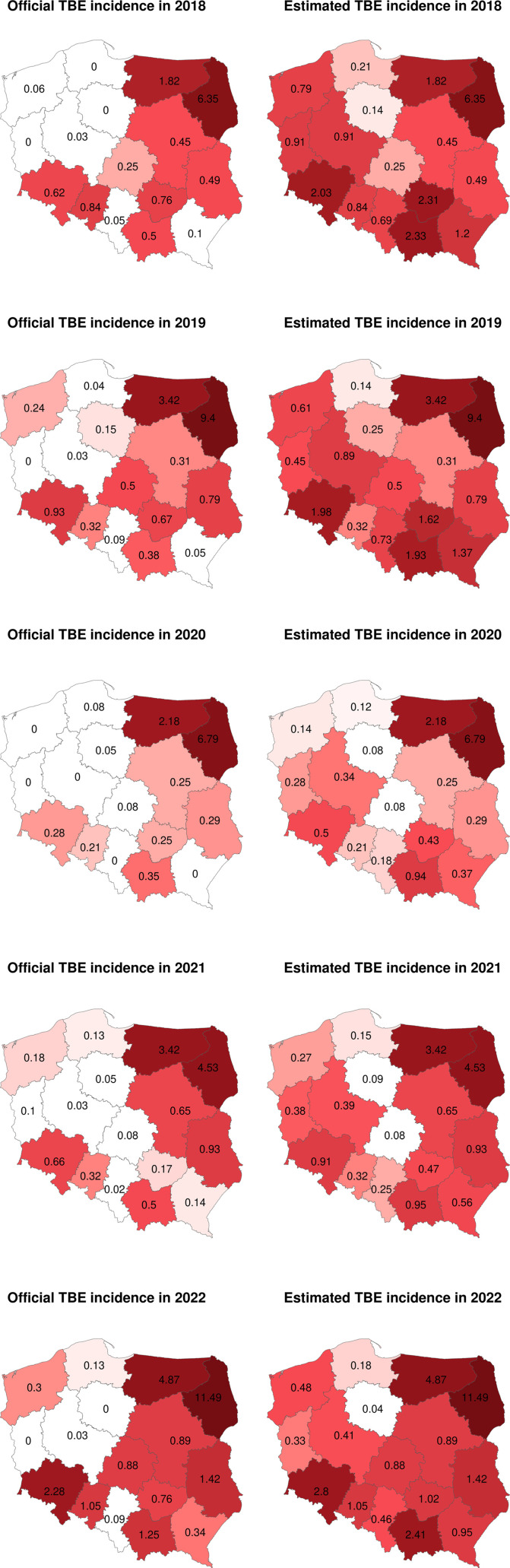
Official and Estimated Morbidity Rates of Tick-Borne Encephalitis by Voivodeship.

We analyzed confirmed infection cases categorized by voivodeship. The results are presented in Table 4 and are depicted in Figure 2. All maps were generated using R software (version 4.3.0) with the raster and ggplot2 packages [14,15]. The study included departments from 10 out of the 16 Polish voivodeships. The southern voivodeships, Małopolskie (31.0%), Świętokrzyskie (24.1%), Dolnośląskie (22.1%), Śląskie (17.3%), and Podkarpackie (17.1%) presented the highest rates of positive samples. Significantly, the Śląskie voivodeship submitted the most samples, totaling 162, contributing to 22.6% of all positive samples in the study. On the contrary, the northern voivodeships showed the lowest positive sample rates: 2.0% in Kujawsko-Pomorskie, 3.0% in Pomorskie, and 7.5% in Zachodniopomorskie. The western voivodeships demonstrated ratios of 15.2% in Wielkopolskie and 17.0% in Lubuskie. What is important to notice, all included departments reported at least one positive case. Figure 3 shows the official morbidity of TBE from 2018 to 2022 in regions as reported by the Polish National Institute of Public Health. Additionally, it shows the estimated morbidity derived from our results, calculated by summing the officially reported cases of TBE with the corresponding percentage of unspecified neuroinfections reported for each voivodeship.

**Table 4:** Cases reported in voivodeships.

Voivodeship	Number of samples	Number of confirmed cases	Percentage of positive samples in the voivodeship	Percentage of all positive samples
Dolnośląskie	145	32	22.1%	25.8%
Kujawsko-Pomorskie	51	1	2.0%	0.8%
Lubuskie	47	8	17.0%	6.5%
Małopolskie	58	18	31.0%	14.5%
Podkarpackie	35	6	17.1%	4.8%
Pomorskie	100	3	3.0%	2.4%
Śląskie	162	28	17.3%	22.6%
Świętokrzyskie	58	14	24.1%	11.3%
Wielkopolskie	66	10	15.2%	8.0%
Zachodniopomorskie	40	3	7.5%	2.4%

## Discussion

### Incidence of TBE in Non-Endemic Regions

The reported incidence of TBE infections in regions of Poland traditionally considered non-endemic is significantly underestimated due to insufficient testing. Historically, detecting the etiology of aseptic central nervous infections has remained challenging, with only 17% of cases having their etiology confirmed between 2004 and 2008 [13]. This trend perpetuated the misguided notion that the risk of TBE was confined to specific regions in Poland. This misconception was challenged by Stefanoff et al. in 2013 [16], who identified 38 new endemic districts in Poland. This pivotal study underscored that the belief that regions were TBE-free was primarily a consequence of low testing rates rather than the actual absence.

Stefanoff et al. [11] pioneered the modeling of TBE risk in Poland and created a TBE risk map that identified potential new risk areas with no officially reported TBE cases. Our findings align with and build upon this foundational work, providing evidence that challenges the previous assumption that only a few regions in Poland are endemic. Specifically, our study identified that 16% of patients presenting with unexplained central nervous system infections in regions previously considered non-endemic were, in fact, diagnosed with TBE. These results support the theoretical assertion that more regions in Poland should be classified as TBE endemic.

Our findings also reflect the theoretical distribution of Stefanoff et al. [12], revealing a coherent regional pattern of positive samples. However, an intriguing contrast emerged because our data demonstrated lower infection ratios in Zachodniopomorskie and Pomorskie than in Wielkopolskie, diverging from the theoretical projection. Climate changes can potentially be responsible for changes in risk distribution within regions [17]. Our results also indicate the detection of new infections throughout the year, even during winter. In essence, our study underscores the omnipresence of TBE risk across all regions of Poland previously considered non-endemic.

### Implications of Clinical Characteristics

The mean age at the time of TBE diagnosis within our study population was 40 years, slightly lower than the average age of 45–50 years reported in the literature [18–20]. It is well established that the severity of TBE infections is mainly associated with age [21]. Interestingly, none of the patients in our study had received prior vaccination against TBE, corresponding to the persistently low uptake of the TBE vaccine observed in Poland. A generally healthy profile was observed among patients diagnosed with TBE, with isolated instances of chronic diseases. Approximately 10% of these patients had a history of Lyme disease, suggesting increased exposure to tick habitats.

All our patients diagnosed with TBE exhibited symptoms of neuroinfection and met at least one laboratory criterion, defined as the presence of IgM or IgG antibodies in blood or cerebrospinal fluid. These criteria differ from the official TBE case confirmation guidelines, which require clinical symptoms alongside both positive IgM and IgG in the blood, or IgM in the cerebrospinal fluid [22]. In our study, we classified patients with only IgM or IgG detected in the blood as TBE cases, as our goal was to conduct an epidemiological study that included as many local hospitals as possible. Many of these hospitals do not perform extended TBE diagnostic procedures, such as waiting for IgG seroconversion, particularly given the lack of specific treatment for TBE [23]. To minimize the risk of false-positive diagnoses, we excluded all patients with other potential causes of elevated IgM, such as autoimmune diseases, neoplasms, or prior vaccination against *Flaviviridae* viruses. Furthermore, TBE virus is currently the only endemic flavivirus in Poland, as epidemiological surveillance has not detected West Nile virus or other flaviviruses as causes of encephalitis cases in the country [24,25].

### Potential Modes of Transmission

Although our study did not involve exhaustive epidemiological investigations into the sources and circumstances of infection among patients, we included a questionnaire to capture information on potential exposure factors. All respondents acknowledged infection’s plausibility within their residence. This phenomenon could be attributed to the climate of Poland, which favors the suitability of TBEV habitat in most of the country [26]. Some patients cited outdoor sports and work-related activities related to natural or forested environments as plausible avenues for exposure. For these individuals, the elevated risk of infection underscores the particular importance of vaccination. In addition, five participants cited the consumption of unpasteurized dairy products as a possible mode of infection. This is in line with the findings by Cisak et al. [27], which indicated the presence of TBEV in the milk of 22% of sheep, 21% of goats, and 11% of cows in Poland.

### Public Health Implications

The implications of our study extend beyond epidemiological insights and are of significant significance for public health strategies. Identifying TBE cases in regions previously considered non-endemic underscores the need for increased vigilance and awareness nationwide. Given the potential severity of TBE, especially in older age groups, our findings emphasize the urgency of public health campaigns to promote TBE vaccination and improve overall vaccination rates. Our results show that the epidemiological situation in Poland does not differ much from that in Lithuania, Slovakia, the Czech Republic, or Austria, where, according to the European Centre for Disease Prevention and Control (ECDC), the entire countries are considered at risk for TBE infections, with a risk level similar to that of endemic regions in Poland [28].

### Limitations and Future Research

Several limitations are inherent to our study. The relatively low sample count from certain voivodeships could have influenced the proportional representation of positive samples in those regions, potentially leading to a misstatement of TBE prevalence. Furthermore, the impact of the COVID-19 pandemic on healthcare systems, including the activity of infectious diseases departments, introduced an external factor that could have affected patient inclusion rates and testing patterns. The absence of comprehensive epidemiological investigations in our study precluded a thorough understanding of the precise sources of infection among patients, leaving room for potential biases in exposure assessments. Our study is also constrained by its focus on specific geographic areas, which may not capture the full extent of underdiagnosed TBE cases. Specifically, we did not included centers located in the areas known as endemic for TBE, as we wanted to evaluate the occurrence in the non-endemic regions. However, we could not exclude that conducting such as analysis could also increase the observed incidence in the endemic regions. However, in those regions the awareness and vigilance of doctors is greater.

Although all of our patients exhibited symptoms consistent with neuroinfection, in some cases, the diagnosis of TBE was based solely on elevated IgM or IgG levels. This alone is insufficient for a definitive diagnosis [22]. However, since TBEV is the only flavivirus endemic to Poland, and we excluded patients vaccinated against flaviviruses while also collecting data on any potential travel-related exposure, we believe that, under these conditions, the specificity (>95%) and sensitivity (>95%) of the ELISA test are adequate for epidemiological purposes [29].

Future research efforts should address these limitations and expand our knowledge of TBE epidemiology. A broader multicenter study involving all departments responsible for treating central nervous system infections across the country could provide a more comprehensive and accurate description of the prevalence of TBE nationwide. Such an approach would allow robust comparisons between voivodeships and yield insights into regional variations. In addition, efforts should be directed towards in-depth investigations into the sources of infections and identification of social risk groups. These efforts could enable the precise targeting of vaccination recommendations, ensuring that the most vulnerable segments of society receive optimal protection.

## Conclusions

In this study, we have dived into the landscape of TBE infections in non-endemic regions of Poland, shedding light on previously unexplored aspects of TBE epidemiology. Our findings underscore the presence of TBE cases in areas historically deemed non-endemic, challenging the prevailing perception and emphasizing the need for increased vigilance throughout the country.
