# Supplementary material for: The impact of serological testing implementation on tick-borne encephalitis detection in Poland
Source: PLoS One. 2025 May 16;20(5):e0323022. doi: 10.1371/journal.pone.0323022 (PMC12084051; doi:10.1371/journal.pone.0323022)
Supplement: Attachment — Submitted filename: TBE_review_response_ver2.docx [file pone.0323022.s001.docx]

# Reviewer 1

1. **In my opinion, the manuscript presents a significant criticality: cases with positive cerebrospinal fluid serology cannot be considered on the same level as those with mere detection of serum antibodies. Furthermore, regarding the positivity in serum for IgM, it is worth noting the a sub-optimal specificity if compared to IgG performance. Before considering these IgM cases as true positives, the authors should have at least observed seroconversion.**

Thank you for highlighting these important aspects of our paper. We agree that the serological methods employed are not entirely sufficient for the clinical diagnosis of TBE. However, our primary aim was to conduct an epidemiological study, not to develop new diagnostic methods for TBE. All of the patients included in our study exhibited symptoms consistent with neuroinfection. It is also important to emphasize that TBEV is the only flavivirus endemic to Poland. According to Prof. Gerhard Dobler (Chapter "Diagnostics of TBEV Infections" in the 7th edition of The TBE Book), ELISA tests demonstrate high sensitivity (up to 99%), but only moderate specificity due to cross-reactivity with other flaviviruses. Nevertheless, specificity can reach up to 97% when testing samples from individuals with no prior exposure to other flaviviruses, as in the case of our research. Furthermore, the comparison of specificity between IgM and IgG tests remains unclear. In the chapter mentioned above, Prof. Dobler notes: “Diagnostic tests for anti-TBEV IgM are usually more specific than IgG tests with regard to cross-reactivity with other flaviviruses (the author’s personal observation).”

It would indeed be valuable to analyze seroconversion over time; however, our study involved many small hospitals. Although this provided a comprehensive view of TBE epidemiology in areas outside of major cities—where many TBE infections occur—it also posed certain limitations. Collaborating with non-academic hospitals required us to adopt simplified diagnostic criteria that all participating institutions could follow, which unfortunately prevented us from observing seroconversion in all patients.

We described the suggested limitations in our Limitations section:

“Although all of our patients exhibited symptoms consistent with neuroinfection, in some cases, the diagnosis of TBE was based solely on elevated IgM or IgG levels. This alone is insufficient for a definitive diagnosis [18]. However, since TBEV is the only flavivirus endemic to Poland, and we excluded patients vaccinated against flaviviruses while also collecting data on any potential travel-related exposure, we believe that, under these conditions, the specificity (>95%) and sensitivity (>95%) of the ELISA test are adequate for epidemiological purposes [23].”

We have also added more description of our diagnostic criteria:

“All our patients diagnosed with TBE exhibited symptoms of neuroinfection and met at least one laboratory criterion, defined as the presence of IgM or IgG antibodies in blood or cerebrospinal fluid. These criteria differ from the official TBE case confirmation guidelines, which require clinical symptoms alongside both positive IgM and IgG in the blood, or IgM in the cerebrospinal fluid [18]. In our study, we classified patients with only IgM or IgG detected in the blood as TBE cases, as our goal was to conduct an epidemiological study that included as many local hospitals as possible. Many of these hospitals do not perform extended TBE diagnostic procedures, such as waiting for IgG seroconversion, particularly given the lack of specific treatment for TBE [19]. To minimize the risk of false-positive diagnoses, we excluded all patients with other potential causes of elevated IgM, such as autoimmune diseases, neoplasms, or prior vaccination against Flaviviridae viruses. Additionally, it is important to note that TBEV is the only flavivirus present in Poland.”

# Reviewer 2

1. **Results: Please mention that the 124 patients with TBEV antibodies were not previously vaccinated against TBEV.**

Thank you for this important suggestion. We have underlined it Result:

“Throughout the study period, from April 1, 2018, to December 31, 2022, we included 766 patients in our analysis, of whom 124 were diagnosed with TBE. None of them were vaccinated against TBE.”

And also in Abstract:

“Among 766 patients, 124 exhibited serum or CSF antibodies against TBEV. None of them were vaccinated against TBE.”

2. **Introduction: The family name Flaviviridae should be in italics.**

We have implemented your suggestion.

3. **Mortality Rate: The mortality rate of 1-5% is often mentioned in the literature, but nowadays it is usually less than 1% in most European countries. Please update this information.**

Thank you for the suggestion. We have clarified that, for the European strain of TBEV, the mortality rate is less than 1%:

“Although some cases of TBE may remain asymptomatic, a significant proportion, ranging from 35% to 58%, can lead to the development of long-term neurological sequelae, with a mortality of European TBEV lower than 1% [3]”

4. **Methods: It is not clear why only patients who received the TBEV vaccine in the last six months were excluded. I would expect that all TBEV vaccinated individuals would be excluded. Please explain.**

Thank you for bringing this to our attention. We agree that prior vaccination is a significant factor in the context of our study. After reevaluating the data, we confirmed that no previously vaccinated patients were included, even those vaccinated more than six months before hospital admission. We have made the following changes to the Methods section:

“Patients who had received a flavivirus vaccination or did not provide their consent to participate were excluded from the study.”

We would also like to emphasize that, although Poland is an endemic region for TBE, only 1–2% of the population is vaccinated against the virus.

5. **Laboratory Analysis: Please specify the kits used for IgG and IgM, including the borderline and positivity values. Also, it would be good to mention the case definition (for example, according to Taba et al., Eur J Neurol. 2017 Oct;24(10):1214-e61. doi: 10.1111/ene.13356.) and then mention in the results/discussion if the newly identified patients indeed meet the case definition.**

Thank you for the suggestion. We have added a paragraph to the discussion addressing our diagnostic criteria. Although these criteria were not sufficient for a clinical diagnosis of TBE, our primary aim was to conduct an epidemiological study:

“All our patients diagnosed with TBE exhibited symptoms of neuroinfection and met at least one laboratory criterion, defined as the presence of IgM or IgG antibodies in blood or cerebrospinal fluid. These criteria differ from the official TBE case confirmation guidelines, which require clinical symptoms alongside both positive IgM and IgG in the blood, or IgM in the cerebrospinal fluid [18]. In our study, we classified patients with only IgM or IgG detected in the blood as TBE cases, as our goal was to conduct an epidemiological study that included as many local hospitals as possible. Many of these hospitals do not perform extended TBE diagnostic procedures, such as waiting for IgG seroconversion, particularly given the lack of specific treatment for TBE [19]. To minimize the risk of false-positive diagnoses, we excluded all patients with other potential causes of elevated IgM, such as autoimmune diseases, neoplasms, or prior vaccination against Flaviviridae viruses. Additionally, it is important to note that TBEV is the only flavivirus present in Poland.”

Additionally, we discussed it in our limitation section:

“Although all of our patients exhibited symptoms consistent with neuroinfection, in some cases, the diagnosis of TBE was based solely on elevated IgM or IgG levels. This alone is insufficient for a definitive diagnosis [18]. However, since TBEV is the only flavivirus endemic to Poland, and we excluded patients vaccinated against flaviviruses while also collecting data on any potential travel-related exposure, we believe that, under these conditions, the specificity (>95%) and sensitivity (>95%) of the ELISA test are adequate for epidemiological purposes [23].”

We have also extended the description of serological diagnosis:

“Serological tests were performed on serum or CSF samples. Patients with clinical symptoms of neuroinfection and a positive result for either IgM or IgG in serum, or IgM or IgG in cerebrospinal fluid (CSF) were diagnosed as TBE patients. To identify anti-TBE immunoglobulins, we used the enzyme-linked immunosorbent assay (ELISA) method, using Virotech ELISA kits (Germany). All analyses were performed at the Immunoserology Laboratory of the Medical University of Białystok. At the same time, patients were required to complete a questionnaire that captured clinical and epidemiological data.”

6. **Clinical Characteristics: It is mentioned here that none of the diagnosed patients had received vaccination against TBEV, but in the methods, it is mentioned that only patients who received the vaccine within the last six months were excluded. Please clarify this discrepancy.**

Thank you for identifying this discrepancy. We have clarified the issue as outlined in your previous question.

7. **Discussion: It would be beneficial to briefly mention the situation in neighboring countries, such as Germany, the Czech Republic, and Slovakia, if this information is available from the literature.**

Thank you for this interesting suggestion. We have included additional information in the discussion and added a reference to the ECDC report:

“Our results show that the epidemiological situation in Poland does not differ much from that in Lithuania, Slovakia, the Czech Republic, or Austria, where, according to the European Centre for Disease Prevention and Control (ECDC), the entire countries are considered at risk for TBE infections, with a risk level similar to that of endemic regions in Poland.”

# Reviewer 3

1. **The authors state that they detected IgM antibodies in serum or CSF samples to confirm TBE infection. However, they do not specify the criteria or definition they followed to confirm TBE as the cause of neuroinfection. This lack of specification raises questions about the accuracy of the diagnosis.**

**The presence of IgM antibodies alone is not a definitive confirmation of TBE. IgM antibodies can yield false-positive results, especially in patients with other health conditions such as autoimmune diseases or mycoplasma infections, which the paper acknowledges were present in some patients. To address this, it would be prudent to follow up with IgM-positive patients to check for seroconversion to IgG antibodies. This follow-up would help rule out false positives and provide a more accurate assessment of TBE incidence.**

Thank you for this important suggestion. We have added the case definition to our Methods section:

“Patients with clinical symptoms of neuroinfection and a positive result for either IgM or IgG in serum, or IgM or IgG in cerebrospinal fluid (CSF) were diagnosed as TBE patients.”

We have also discussed the difference between our case definition and official recommendations:

“All our patients diagnosed with TBE exhibited symptoms of neuroinfection and met at least one laboratory criterion, defined as the presence of IgM or IgG antibodies in blood or cerebrospinal fluid. These criteria differ from the official TBE case confirmation guidelines, which require clinical symptoms alongside both positive IgM and IgG in the blood, or IgM in the cerebrospinal fluid [18]. In our study, we classified patients with only IgM or IgG detected in the blood as TBE cases, as our goal was to conduct an epidemiological study that included as many local hospitals as possible. Many of these hospitals do not perform extended TBE diagnostic procedures, such as waiting for IgG seroconversion, particularly given the lack of specific treatment for TBE [19]. To minimize the risk of false-positive diagnoses, we excluded all patients with other potential causes of elevated IgM, such as autoimmune diseases, neoplasms, or prior vaccination against Flaviviridae viruses. Additionally, it is important to note that TBEV is the only flavivirus present in Poland.”

We have also discussed the limitations of our study caused by this case definition:

“Although all of our patients exhibited symptoms consistent with neuroinfection, in some cases, the diagnosis of TBE was based solely on elevated IgM or IgG levels. This alone is insufficient for a definitive diagnosis [18]. However, since TBEV is the only flavivirus endemic to Poland, and we excluded patients vaccinated against flaviviruses while also collecting data on any potential travel-related exposure, we believe that, under these conditions, the specificity (>95%) and sensitivity (>95%) of the ELISA test are adequate for epidemiological purposes [23].”
