# Supplementary material for: The impact of serological testing implementation on tick-borne encephalitis detection in Poland
Source: PLoS One. 2025 May 16;20(5):e0323022. doi: 10.1371/journal.pone.0323022 (PMC12084051; doi:10.1371/journal.pone.0323022)
Supplement: Attachment — Submitted filename: review2_response.docx [file pone.0323022.s002.docx]

Thank you for your thorough review and valuable suggestions. We have addressed each of your comments in detail below.

**1. The Factors of socio-demography: what role does it play in the prevalence or incidence of TBE in the region? What were the findings of previous study and how did that influence or affect the current study. Is there a correlation in result or is this a fresh gap that has been identified?**

Thank you for raising this important question. In the literature, TBE risk is generally not associated with socio-demographic factors, as the primary determinants of risk are environmental conditions and activities that increase tick exposure. According to the most recent Annual Epidemiological Report from the European Centre for Disease Prevention and Control (ECDC), TBE cases are more frequently reported among men (male-to-female ratio: 1.5:1), with the highest incidence observed in the 45–64 age group (<https://www.ecdc.europa.eu/en/publications-data/tick-borne-encephalitis-annual-epidemiological-report-2022>)

Previous studies indicate that these demographic differences are likely driven by patterns of occupational and recreational exposure. Specifically, spending over 10 hours per week in forests—either for work or leisure—is a known risk factor for TBE in endemic regions ([10.1371/journal.pone.0045511](https://doi.org/10.1371/journal.pone.0045511))

We have incorporated this context into the Introduction section of our manuscript as follows:

“In Europe, TBE cases are more frequently reported among men, particularly in the 45–64 age group. This pattern is likely explained by increased exposure to forested environments during work or recreational activities, as spending more than 10 hours per week in forests within endemic regions is a recognized risk factor for TBE infection.”

**2. The introduction fails to establish the demography of the target audience as well as the epidemiology .How does other studies done influence the study at hand. Were there critical literature reviews and how does it affect the current finding?
What other literature shows the gap?**

Thank you for this important comment. We have added a new paragraph to the Introduction, which describes the demographic aspects and integrates previous theoretical research that serves as the foundation for our empirical study.

“Currently, two regions in Poland—Podlaskie and Warmińsko-Mazurskie voivodeships—are recognized as endemic for TBE. These regions are home to over 2.5 million people, with approximately 30% of the land area covered by forests. As popular tourist destinations, these areas attract around 1.5 million visitors annually, further increasing potential exposure to ticks. However, a theoretical analysis conducted by Stefanoff et al. suggested that the entire territory of Poland could be considered endemic for TBE. To date, these theoretical findings have not been empirically confirmed.”

**3. While discussing the Objective of the study, what is the relationship between the Independent and dependent variables. How does the independent variable affect the outcome of the study?**

Thank you for this thoughtful observation. In our study, we do not operate with traditional independent and dependent variables, as our primary objective is to estimate the proportion of neuroinfections of unknown origin that are, in fact, TBE infections. This approach is intended to provide a more accurate representation of the true TBE incidence in Poland. To emphasize this, we have revised our "Aim of the Study" as follows:

“This study aims to determine the true prevalence of TBE in traditionally non-endemic regions of Poland by conducting systematic serological analyses of serum and cerebrospinal fluid samples obtained from patients presenting with unexplained neuroinfections. The ultimate goals are to identify emerging TBE hotspots and assess potential underreporting by comparing the findings with official statistics.”

**4. The study fails to elucidate the reason behind the choice of the Methodology used. What is the justification for the study location? How did you arrive at the study site. Why chose 29 departments and not 20? Was there a set of criteria that were used? What is the specific demography of the study site that warranted the choice?**

Thank you for raising this point. We have expanded the description in the Methods section to address all your questions:

“We invited all departments treating neuroinfections from regions identified as potentially having a higher-than-reported TBE incidence to participate in the study. Ultimately, 29 departments agreed to take part, including infectious disease units and neurology departments specializing in diagnosing and treating neuroinfections. These centers were strategically located in areas with limited official reporting of TBE cases, but preliminary data indicated the potential presence of undiagnosed TBE cases. Furthermore, these departments had the capacity to perform the serological tests required for this study. The Department of Infectious Diseases and Neuroinfections at the Medical University of Białystok oversaw and coordinated the participation of all included departments.”

**5. While analyzing the discussion and the significant finding, it was not clear if it correlated with the Significant finding in Literature Review.
The study has not defined the limit of study limitation .**

Thank you for this valuable comment. We have revised the Discussion section to better contextualize our findings within the theoretical framework proposed by Stefanoff et al. (10.1371/journal.pone.0045511) and to address the study limitations.

“Stefanoff et al. [10] pioneered the modeling of TBE risk in Poland and created a TBE risk map that identified potential new risk areas with no officially reported TBE cases. Our findings align with and build upon this foundational work, providing evidence that challenges the previous assumption that only a few regions in Poland are endemic. Specifically, our study identified that 16% of patients presenting with unexplained central nervous system infections in regions previously considered non-endemic were, in fact, diagnosed with TBE. These results support Stefanoff et al.’s assertion that more regions in Poland should be classified as TBE endemic.”

We have also extended one paragraph in Limitations

“Several limitations are inherent to our study. The relatively low sample count from certain voivodeships could have influenced the proportional representation of positive samples in those regions, potentially leading to a misstatement of TBE prevalence. Furthermore, the impact of the COVID-19 pandemic on healthcare systems, including the activity of infectious diseases departments, introduced an external factor that could have affected patient inclusion rates and testing patterns. The absence of comprehensive epidemiological investigations in our study precluded a thorough understanding of the precise sources of infection among patients, leaving room for potential biases in exposure assessments. Our study is also constrained by its focus on specific geographic areas, which may not capture the full extent of underdiagnosed TBE cases.”
