# Supplementary material for: The impact of serological testing implementation on tick-borne encephalitis detection in Poland
Source: PLoS One. 2025 May 16;20(5):e0323022. doi: 10.1371/journal.pone.0323022 (PMC12084051; doi:10.1371/journal.pone.0323022)
Supplement: Attachment — Submitted filename: Review PLOS.docx [file pone.0323022.s003.docx]

Review

Aims: What are “official statistics”?

Methods: “How did you know that the regions are having “potentially higher then reported TBE incidence rates”?

How many departments did you contact? Which ones were excluded and why did you not take the “risk areas” as a positive control?

April 1^st^, December 31^st^

Explain CFS in the first sentence of “Laboratory analysis” not later

Can you give details of how Igg and IgM were measured? What was the cut off titre? Is it a commercial kit? Was the method unis ono in all laboratories?

Results

Were 124 included in the study, previously detected as tbe cases or did you detect that? Why did the clinicians not check for tbe themselves then?

Table 4 should be a map

The study would benefit from a statistical analysis. For someone not from Poland it is hard to understand which departments were selected, why and which departments are TBE hot spots. Geografical explanation would benefit the discussion to explain possible tbe movement. Further, can it be excluded in these patients that they did not travel abroad or were in other departmens before or even hot spot areas?

Which also does not come out from this paper is what the authors consider a “risk area”, how many cases per habitants per year? Showing absence and presence of tbe does not define areas at risk.

Did the authors show only areas that were positive for tbe, were there also departments that are indeed without positive cases?
