# Supplementary material for: The impact of serological testing implementation on tick-borne encephalitis detection in Poland
Source: PLoS One. 2025 May 16;20(5):e0323022. doi: 10.1371/journal.pone.0323022 (PMC12084051; doi:10.1371/journal.pone.0323022)
Supplement: Attachment — Submitted filename: PLOS_review3.docx [file pone.0323022.s004.docx]

**Reviewer 2**

Thank you very much for your thorough review and constructive feedback. We appreciate your detailed comments.

**Reviewer 3**

Thank you very much for your comprehensive suggestions and thoughtful insights. We believe that implementing these changes has significantly improved our manuscript's clarity and scientific rigor.

1. “However, the issue of relying on IgM positivity without IgG seroconversion remains a gap, what leaves room for potential diagnostic inaccuracies.”

Thank you for raising this important point about IgM/IgG diagnostics. We have carefully considered the potential limitations of relying on IgM positivity and have taken several steps to ensure diagnostic accuracy. As we discuss in the manuscript, TBEV is the only Flavivirus in Poland that causes CNS infections. However, in response to your valid concern, we have expanded this section of the Discussion and added key references documenting the absence of other Flaviviridae infections in Poland:

“To minimize the risk of false-positive diagnoses, we excluded all patients with other potential causes of elevated IgM, such as autoimmune diseases, neoplasms, or prior vaccination against *Flaviviridae* viruses. Furthermore, TBE virus is currently the only endemic flavivirus in Poland, as epidemiological surveillance has not detected West Nile virus or other flaviviruses as causes of encephalitis cases in the country [24,25].”

**Reviewer 5**

Thank you for your thorough and insightful review. We appreciate your detailed suggestions for improving our manuscript. We have carefully considered each point and provided our responses below.

1.“This new text does not fit well into the introduction. Please consider rewriting it.”

Thank you for this helpful observation regarding the text flow. We have restructured the Introduction to improve its coherence by merging related content about therapies and vaccination into a single, cohesive paragraph and creating a separate, dedicated paragraph for epidemiological information.

2.“Aim of the study: In relation to this new text, please consider revising or removing the term "true prevalence," as it may not be appropriate given the study design and its limitations.”

Thank you for this suggestion. We have changed it as follows:

“This study aims to evaluate the occurrence and distribution of TBE in traditionally non-endemic regions of Poland by conducting systematic serological analyses of serum and cerebrospinal fluid samples obtained from patients presenting with unexplained neuroinfections. The ultimate goals are to identify emerging TBE hotspots and assess potential underreporting by comparing the findings with official statistics.”

3.“Methods/included centers: Could you provide information about the centers or areas not covered in your study? Are there any data limitations related to this point?”

Thank you for this important question about geographic coverage. We have elaborated on this limitation in our revised manuscript:

“Our study has geographic limitations, as it focused primarily on regions traditionally considered non-endemic for TBE. We intentionally excluded centers from known endemic areas to specifically evaluate TBE occurrence in presumed non-endemic regions. While this approach served our primary research objective, it means we cannot provide a comprehensive national picture of TBE incidence. It's possible that applying similar systematic testing protocols in endemic regions could reveal even higher incidence rates than currently reported, although healthcare providers in these areas typically maintain higher awareness and vigilance for TBE cases”

4.“Laboratory analysis: Why was a TBEV neutralization test not included to confirm the positive samples? Obtaining results from this test would have provided valuable insights into the level of neutralizing antibodies in the patients. However, I recognize that there may have been limitations, such as limited sample volume, which prevented this analysis.”

Thank you for this important point. We did not perform neutralization tests for several reasons:

1. Our primary goal was to conduct a large-scale epidemiological study involving multiple local hospitals. Adding neutralization testing would have significantly increased the complexity and cost of the study.
2. Given the absence of other endemic flaviviruses in Poland during the study period (as confirmed by national surveillance data showing no detection of West Nile virus or other flaviviruses), and our careful exclusion of patients with prior flavivirus vaccinations or potential cross-reactive conditions, we considered the specificity of ELISA testing sufficient for our epidemiological purposes.
3. The study's focus was on identifying potential TBE cases in non-endemic regions to demonstrate the need for enhanced surveillance, rather than conducting detailed serological characterization of each case.

5. “Discussion: Paragraph 3:Include a reference to the specific ELISA test used in this study along with reference 27. The specificity and sensitivity of the test should take into account its cross-reactivity with related flaviviruses. Limitations and future research: Please include information regarding cross-reactions in the ELISA test for other flaviviruses. How can it be confirmed that this is caused by TBEV? It could potentially be other unknown flaviviruses. Additionally, provide more details about related or possible travel-related cases, particularly for patients who are only IgG positive.”

Thank you for these important points about testing specificity and potential cross-reactivity. Our approach to ensuring accurate diagnosis was comprehensive. To minimize the risk of false-positive diagnoses, we excluded all patients with other potential causes of elevated IgM, such as autoimmune diseases, neoplasms, or prior vaccination against *Flaviviridae* viruses. Furthermore, TBE virus is currently the only endemic flavivirus in Poland, as epidemiological surveillance has not detected West Nile virus or other flaviviruses as causes of encephalitis cases in the country. We have added the description and references:

“To minimize the risk of false-positive diagnoses, we excluded all patients with other potential causes of elevated IgM, such as autoimmune diseases, neoplasms, or prior vaccination against Flaviviridae viruses. Furthermore, TBE virus is currently the only endemic flavivirus in Poland, as epidemiological surveillance has not detected West Nile virus or other flaviviruses as causes of encephalitis cases in the country [24,25].”

Regarding travel-related cases, we conducted detailed interviews with all patients about possible sources of infection. Only 3 of them traveled abroad – to Hungary, Corfu, and Croatia, as shown in Table 3. We have improved this description in the manuscript:

“Additionally, 11 patients were suspected of contracting the infection during travel (3 of them abroad – Hungary, Corfu or Croatia), and an additional five patients reported consuming unpasteurized dairy products. A comprehensive breakdown of these potential infection sources is provided in Table 3.”

**Reviewer 6**

Thank you very much for all your constructive comments. We have addressed each point and implemented the corresponding changes in the manuscript, as detailed below.

1.“Aims: What are “official statistics”?”

Regarding your question about 'official statistics', we have extended and clarified this explanation as follows:

“The ultimate goals are to identify emerging TBE hotspots and assess potential underreporting by comparing the findings with official statistics from the Polish National Institute of Public Health's mandatory surveillance system.”

2.“Methods: “How did you know that the regions are having “potentially higher then reported TBE incidence rates”?”

Thank you for this important point. We have clarified our methodology in the Methods section:

“We invited all departments treating neuroinfections from regions identified as potentially having a higher-than-reported TBE incidence to participate in the study. This selection was based on previous theoretical modeling studies by Stefanoff et al. [11], which identified potential high-risk areas through analysis of environmental factors conducive to TBEV transmission”

3.How many departments did you contact? Which ones were excluded and why did you not take the “risk areas” as a positive control?

Thank you for these important questions about study site selection. We contacted all departments treating neuroinfections in non-endemic regions of Poland. Out of these, 29 departments agreed to participate, as described in our Methods section:

“We invited all departments treating neuroinfections from regions identified as potentially having a higher-than-reported TBE incidence to participate in the study.”

Regarding the exclusion of 'risk areas' (endemic regions) as control groups, this decision was based on several methodological considerations:

- Our primary objective was to evaluate the occurrence of TBE in traditionally non-endemic regions where testing for TBEV is not routinely performed. In contrast, endemic regions already have established testing protocols and higher awareness among medical professionals.
- Including endemic regions as controls would not have served our main research goal, which was to assess potential underdiagnosis in areas where TBE is not typically suspected. In endemic regions, patients are already widely tested for this etiology.
- This was designed as an epidemiological study specifically focusing on detecting TBE cases among patients with unexplained neuroinfections in presumed non-endemic areas, rather than a comprehensive national survey of TBE incidence.

We have acknowledged this limitation in our manuscript:

“Our study has geographic limitations, as it focused primarily on regions traditionally considered non-endemic for TBE. We intentionally excluded centers from known endemic areas to specifically evaluate TBE occurrence in presumed non-endemic regions. While this approach served our primary research objective, it means we cannot provide a comprehensive national picture of TBE incidence. It's possible that applying similar systematic testing protocols in endemic regions could reveal even higher incidence rates than currently reported, although healthcare providers in these areas typically maintain higher awareness and vigilance for TBE cases”

4.“April 1st, December 31st

Thank you for this suggestion about date formatting. However, we have chosen to maintain the format 'April 1, 2018, and December 31, 2022' rather than using ordinal indicators (1st, 31st) because this format aligns with the widely accepted scientific writing conventions used in medical literature.

5.“Explain CFS in the first sentence of “Laboratory analysis” not later”

Thank you for finding this mistake. We have corrected it.

6.Can you give details of how Igg and IgM were measured? What was the cut off titre? Is it a commercial kit? Was the method unis ono in all laboratories?

Thank you for these important questions about our testing methodology. All serological analyses were conducted using a standardized approach. We used the commercially available Virotech ELISA kit (Germany) for detecting both IgG and IgM antibodies against TBEV, as described:

“To identify anti-TBE immunoglobulins, we used the enzyme-linked immunosorbent assay (ELISA) method, using Virotech ELISA kits (Germany).”

All analyses were centralized and performed at a single location - the Immunoserology Laboratory of the Medical University of Białystok to ensure consistency and eliminate inter-laboratory variability. This centralized testing approach eliminated potential technical discrepancies that could arise from multiple testing sites, as described:

“All analyses were performed at the Immunoserology Laboratory of the Medical University of Białystok.”

7.Were 124 included in the study, previously detected as tbe cases or did you detect that? Why did the clinicians not check for tbe themselves then?

Thank you for this important question about case identification. The 124 TBE cases were newly detected through our study, not previously diagnosed cases. Prior to our study, TBE testing was not routinely performed in regions considered non-endemic. This was due to the prevailing belief that TBE was limited to certain endemic regions in Poland, combined with the absence of specific antiviral treatment for TBE and limited awareness of TBE risk in these areas.

Our study was initiated based on theoretical modeling work by Stefanoff et al., which suggested that TBE risk might extend beyond traditionally recognized endemic regions. As we described in the paper, we included patients who “exhibited clinical indicators suggestive of TBE, such as inflammation of the central nervous system (manifesting as meningitis, meningoencephalitis, encephalomyelitis or encephaloradiculitis) but the specific type of neuroinfection was not confirmed. Initially, these patients were classified within epidemiological surveillance as cases of unspecified viral meningitis (coded A87, B00.3, B02.1), unspecified meningitis (coded G03), other viral-specific encephalitis (coded A83, A85, B00.4, B02.0, B25.8), or unspecified encephalitis (coded A86, G04.8, G04.9).”

8.Table 4 should be a map

Thank you for this suggestion, Figure 2 depicts data from Table 4.

9.The study would benefit from a statistical analysis. For someone not from Poland it is hard to understand which departments were selected, why and which departments are TBE hot spots. Geografical explanation would benefit the discussion to explain possible tbe movement. Further, can it be excluded in these patients that they did not travel abroad or were in other departmens before or even hot spot areas?

Thank you for these important points about geographical analysis and travel history. Our paper includes several visualization tools to help readers understand the geographical and epidemiological context of TBE in Poland. Figure 2 shows the voivodeships participating in our research and their respective percentages of positive cases, while Figures 3A-E present the official reported epidemiological situation across regions. These visualizations provide a clear picture of both our study's geographic scope and the broader epidemiological context.

We carefully documented potential exposure sources, including travel. As shown in Table 3, only 11 of our 124 detected TBE patients (less than 10%) reported travel history - 3 abroad and 8 to other regions of Poland. This means that for over 90% of cases, there was a clear connection between infection and place of residence. Regarding travel as a potential confounder, this remains epidemiologically significant even if some patients acquired TBE in other regions. The mobility of populations means that healthcare providers in all regions should maintain awareness of TBE during their diagnostic process, regardless of their location's endemic status.

10.Which also does not come out from this paper is what the authors consider a “risk area”, how many cases per habitants per year? Showing absence and presence of tbe does not define areas at risk.

Thank you for this important point about defining risk areas. We have added clarification in the Methods section that we used the World Health Organization's (WHO) standardized definition of TBE risk areas, which defines a risk area as having ≥5 cases/100,000 population/year (<https://cdn.who.int/media/docs/default-source/immunization/position_paper_documents/tick-borne-encephalitis/who-pp-tbe-2011-summary.pdf?sfvrsn=de01b1fe_2>)

11.Did the authors show only areas that were positive for tbe, were there also departments that are indeed without positive cases?

Thank you for this important question. All departments participating in our study identified at least one positive TBE case during the study period, as we have now clarified in the Results section: “What is important to notice, all included departments reported at least one positive case.” This finding is particularly significant for regions like Lubuskie voivodeship, which had no officially reported TBE cases prior to our study, as shown in Figure 3.
