# Supplementary material for: The impact of serological testing implementation on tick-borne encephalitis detection in Poland
Source: PLoS One. 2025 May 16;20(5):e0323022. doi: 10.1371/journal.pone.0323022 (PMC12084051; doi:10.1371/journal.pone.0323022)
Supplement: Peer review history

# Author comment: Author response to Decision Letter 0

**DOI:** 10.1371/journal.pone.0323022.r001

23 Jan 2024

# Peer review: Decision Letter 0

**DOI:** 10.1371/journal.pone.0323022.r002

**Authors:** Oluyinka Ajibola Iyiola

21 Aug 2024

PONE-D-24-01666The Impact of Serological Testing Implementation on Tick-Borne Encephalitis Detection in PolandPLOS ONE

Dear Dr. Wawrzuta,

Thank you for submitting your manuscript to PLOS ONE. After careful consideration, we feel that it has merit but does not fully meet PLOS ONE’s publication criteria as it currently stands. Therefore, we invite you to submit a revised version of the manuscript that addresses the points raised during the review process.

Please submit your revised manuscript by Oct 05 2024 11:59PM. If you will need more time than this to complete your revisions, please reply to this message or contact the journal office at plosone@plos.org . When you're ready to submit your revision, log on to https://www.editorialmanager.com/pone/ and select the 'Submissions Needing Revision' folder to locate your manuscript file.

Please include the following items when submitting your revised manuscript:

- A rebuttal letter that responds to each point raised by the academic editor and reviewer(s). You should upload this letter as a separate file labeled 'Response to Reviewers'.
- A marked-up copy of your manuscript that highlights changes made to the original version. You should upload this as a separate file labeled 'Revised Manuscript with Track Changes'.
- An unmarked version of your revised paper without tracked changes. You should upload this as a separate file labeled 'Manuscript'.

If you would like to make changes to your financial disclosure, please include your updated statement in your cover letter. Guidelines for resubmitting your figure files are available below the reviewer comments at the end of this letter.

If applicable, we recommend that you deposit your laboratory protocols in protocols.io to enhance the reproducibility of your results. Protocols.io assigns your protocol its own identifier (DOI) so that it can be cited independently in the future. For instructions see: https://journals.plos.org/plosone/s/submission-guidelines#loc-laboratory-protocols . Additionally, PLOS ONE offers an option for publishing peer-reviewed Lab Protocol articles, which describe protocols hosted on protocols.io. Read more information on sharing protocols at https://plos.org/protocols?utm_medium=editorial-email&utm_source=authorletters&utm_campaign=protocols .

We look forward to receiving your revised manuscript.

Kind regards,

Oluyinka Ajibola Iyiola

Academic Editor

PLOS ONE

Journal requirements:

When submitting your revision, we need you to address these additional requirements.

1. Please ensure that your manuscript meets PLOS ONE's style requirements, including those for file naming. The PLOS ONE style templates can be found at

https://journals.plos.org/plosone/s/file?id=wjVg/PLOSOne_formatting_sample_main_body.pdf and

https://journals.plos.org/plosone/s/file?id=ba62/PLOSOne_formatting_sample_title_authors_affiliations.pdf

2. Please provide additional details regarding participant consent. In the ethics statement in the Methods and online submission information, please ensure that you have specified (1) whether consent was informed and (2) what type you obtained (for instance, written or verbal, and if verbal, how it was documented and witnessed). If your study included minors, state whether you obtained consent from parents or guardians. If the need for consent was waived by the ethics committee, please include this information.

If you are reporting a retrospective study of medical records or archived samples, please ensure that you have discussed whether all data were fully anonymized before you accessed them and/or whether the IRB or ethics committee waived the requirement for informed consent. If patients provided informed written consent to have data from their medical records used in research, please include this information.

3. We note that the grant information you provided in the ‘Funding Information’ and ‘Financial Disclosure’ sections do not match.

When you resubmit, please ensure that you provide the correct grant numbers for the awards you received for your study in the ‘Funding Information’ section.

4. We note that you have indicated that there are restrictions to data sharing for this study. PLOS only allows data to be available upon request if there are legal or ethical restrictions on sharing data publicly. For more information on unacceptable data access restrictions, please see http://journals.plos.org/plosone/s/data-availability#loc-unacceptable-data-access-restrictions.

Before we proceed with your manuscript, please address the following prompts:

a) If there are ethical or legal restrictions on sharing a de-identified data set, please explain them in detail (e.g., data contain potentially identifying or sensitive patient information, data are owned by a third-party organization, etc.) and who has imposed them (e.g., a Research Ethics Committee or Institutional Review Board, etc.). Please also provide contact information for a data access committee, ethics committee, or other institutional body to which data requests may be sent.

b) If there are no restrictions, please upload the minimal anonymized data set necessary to replicate your study findings to a stable, public repository and provide us with the relevant URLs, DOIs, or accession numbers. For a list of recommended repositories, please see

https://journals.plos.org/plosone/s/recommended-repositories. You also have the option of uploading the data as Supporting Information files, but we would recommend depositing data directly to a data repository if possible.

We will update your Data Availability statement on your behalf to reflect the information you provide.

5. Please include a separate caption for each figure in your manuscript.

6. We note that Figures 2, 3A, 3B, 3C, 3D and 3E in your submission contain [map/satellite] images which may be copyrighted. All PLOS content is published under the Creative Commons Attribution License (CC BY 4.0), which means that the manuscript, images, and Supporting Information files will be freely available online, and any third party is permitted to access, download, copy, distribute, and use these materials in any way, even commercially, with proper attribution. For these reasons, we cannot publish previously copyrighted maps or satellite images created using proprietary data, such as Google software (Google Maps, Street View, and Earth). For more information, see our copyright guidelines: http://journals.plos.org/plosone/s/licenses-and-copyright.

We require you to either (1) present written permission from the copyright holder to publish these figures specifically under the CC BY 4.0 license, or (2) remove the figures from your submission:

a. You may seek permission from the original copyright holder of Figures 2, 3A, 3B, 3C, 3D and 3E to publish the content specifically under the CC BY 4.0 license. 

We recommend that you contact the original copyright holder with the Content Permission Form (http://journals.plos.org/plosone/s/file?id=7c09/content-permission-form.pdf) and the following text:

“I request permission for the open-access journal PLOS ONE to publish XXX under the Creative Commons Attribution License (CCAL) CC BY 4.0 (http://creativecommons.org/licenses/by/4.0/). Please be aware that this license allows unrestricted use and distribution, even commercially, by third parties. Please reply and provide explicit written permission to publish XXX under a CC BY license and complete the attached form.”

Please upload the completed Content Permission Form or other proof of granted permissions as an "Other" file with your submission.

In the figure caption of the copyrighted figure, please include the following text: “Reprinted from [ref] under a CC BY license, with permission from [name of publisher], original copyright [original copyright year].”

b. If you are unable to obtain permission from the original copyright holder to publish these figures under the CC BY 4.0 license or if the copyright holder’s requirements are incompatible with the CC BY 4.0 license, please either i) remove the figure or ii) supply a replacement figure that complies with the CC BY 4.0 license. Please check copyright information on all replacement figures and update the figure caption with source information. If applicable, please specify in the figure caption text when a figure is similar but not identical to the original image and is therefore for illustrative purposes only.

The following resources for replacing copyrighted map figures may be helpful:

USGS National Map Viewer (public domain): http://viewer.nationalmap.gov/viewer/

The Gateway to Astronaut Photography of Earth (public domain): http://eol.jsc.nasa.gov/sseop/clickmap/

Maps at the CIA (public domain): https://www.cia.gov/library/publications/the-world-factbook/index.html and https://www.cia.gov/library/publications/cia-maps-publications/index.html

NASA Earth Observatory (public domain): http://earthobservatory.nasa.gov/

Landsat: http://landsat.visibleearth.nasa.gov/

USGS EROS (Earth Resources Observatory and Science (EROS) Center) (public domain): http://eros.usgs.gov/#

Natural Earth (public domain): http://www.naturalearthdata.com/

7. Please review your reference list to ensure that it is complete and correct. If you have cited papers that have been retracted, please include the rationale for doing so in the manuscript text, or remove these references and replace them with relevant current references. Any changes to the reference list should be mentioned in the rebuttal letter that accompanies your revised manuscript. If you need to cite a retracted article, indicate the article’s retracted status in the References list and also include a citation and full reference for the retraction notice.

[Note: HTML markup is below. Please do not edit.]

Reviewers' comments:

Reviewer's Responses to Questions

**Comments to the Author**

1. Is the manuscript technically sound, and do the data support the conclusions?

The manuscript must describe a technically sound piece of scientific research with data that supports the conclusions. Experiments must have been conducted rigorously, with appropriate controls, replication, and sample sizes. The conclusions must be drawn appropriately based on the data presented. 

Reviewer #1: Partly

Reviewer #2: Yes

Reviewer #3: Yes

**********

2. Has the statistical analysis been performed appropriately and rigorously? 

Reviewer #1: No

Reviewer #2: N/A

Reviewer #3: Yes

**********

3. Have the authors made all data underlying the findings in their manuscript fully available?

The PLOS Data policy requires authors to make all data underlying the findings described in their manuscript fully available without restriction, with rare exception (please refer to the Data Availability Statement in the manuscript PDF file). The data should be provided as part of the manuscript or its supporting information, or deposited to a public repository. For example, in addition to summary statistics, the data points behind means, medians and variance measures should be available. If there are restrictions on publicly sharing data—e.g. participant privacy or use of data from a third party—those must be specified.

Reviewer #1: Yes

Reviewer #2: Yes

Reviewer #3: Yes

**********

4. Is the manuscript presented in an intelligible fashion and written in standard English?

PLOS ONE does not copyedit accepted manuscripts, so the language in submitted articles must be clear, correct, and unambiguous. Any typographical or grammatical errors should be corrected at revision, so please note any specific errors here.

Reviewer #1: Yes

Reviewer #2: Yes

Reviewer #3: Yes

**********

5. Review Comments to the Author

Please use the space provided to explain your answers to the questions above. You may also include additional comments for the author, including concerns about dual publication, research ethics, or publication ethics. (Please upload your review as an attachment if it exceeds 20,000 characters)

Reviewer #1: This study aims to assess the incidence of tick-borne encephalitis (TBE) in non-endemic regions of Poland by analyzing serum or cerebrospinal fluid (CSF) samples from patients with neuroinfections of unknown origin.

In my opinion, the manuscript presents a significant criticality: cases with positive cerebrospinal fluid serology cannot be considered on the same level as those with mere detection of serum antibodies. Furthermore, regarding the positivity in serum for IgM, it is worth noting the a sub-optimal specificity if compared to IgG performance. Before considering these IgM cases as true positives, the authors should have at least observed seroconversion.

Reviewer #2: Zajkowska et al. submitted a manuscript titled: “The impact of serological testing implementation on tick-borne encephalitis detection in Poland” for peer review in PLoS One. The manuscript is of high interest as it identifies novel tick-borne encephalitis virus (TBEV) endemic regions in Poland, which were previously considered to be non-endemic. More importantly, it identifies that many patients from these regions who were previously diagnosed with cases of aseptic meningitis/encephalitis of unknown origin actually represent cases of TBE. This is an important discovery that deserves publication.

Minor comments:

1. Abstract – Results: Please mention that the 124 patients with TBEV antibodies were not previously vaccinated against TBEV.

2. Introduction: The family name Flaviviridae should be in italics.

3. Mortality Rate: The mortality rate of 1-5% is often mentioned in the literature, but nowadays it is usually less than 1% in most European countries. Please update this information.

4. Methods: It is not clear why only patients who received the TBEV vaccine in the last six months were excluded. I would expect that all TBEV vaccinated individuals would be excluded. Please explain.

5. Methods – Laboratory Analysis: Please specify the kits used for IgG and IgM, including the borderline and positivity values. Also, it would be good to mention the case definition (for example, according to Taba et al., Eur J Neurol. 2017 Oct;24(10):1214-e61. doi: 10.1111/ene.13356.) and then mention in the results/discussion if the newly identified patients indeed meet the case definition.

5. Serologic and Cerebrospinal Fluid Analysis: “… the presence of TBEV-specific IgM antibodies…”.

7. Clinical Characteristics: It is mentioned here that none of the diagnosed patients had received vaccination against TBEV, but in the methods, it is mentioned that only patients who received the vaccine within the last six months were excluded. Please clarify this discrepancy.

8. Discussion: It would be beneficial to briefly mention the situation in neighboring countries, such as Germany, the Czech Republic, and Slovakia, if this information is available from the literature.

Reviewer #3: The paper titled "The Impact of Serological Testing Implementation on Tick-Borne Encephalitis Detection in Poland" is a well-written and thorough examination of TBE incidence in non-endemic regions of Poland. The study's methodology, data collection, and analysis are robust and contribute significantly to understanding the underreporting of TBE in these areas.

The inclusion of 29 neuroinfection-specialized departments from various non-endemic regions provides a broad and representative sample of the population at risk. This extensive data collection enhances the reliability and validity of the study's findings. The paper effectively presents its results, showing significant regional differences in TBE incidence. This clear delineation helps highlight areas where public health interventions might be most needed. The study underscores the underreporting of TBE in Poland, which is a crucial public health issue. By identifying the incidence of TBE in traditionally non-endemic regions, the study provides a foundation for better surveillance and awareness programs.

While the study is generally sound, there is a notable concern regarding the method used to confirm TBE infection:

- The authors state that they detected IgM antibodies in serum or CSF samples to confirm TBE infection. However, they do not specify the criteria or definition they followed to confirm TBE as the cause of neuroinfection. This lack of specification raises questions about the accuracy of the diagnosis.

- The presence of IgM antibodies alone is not a definitive confirmation of TBE. IgM antibodies can yield false-positive results, especially in patients with other health conditions such as autoimmune diseases or mycoplasma infections, which the paper acknowledges were present in some patients. To address this, it would be prudent to follow up with IgM-positive patients to check for seroconversion to IgG antibodies. This follow-up would help rule out false positives and provide a more accurate assessment of TBE incidence.

Despite the concern regarding the confirmation method for TBE infection, the study is a valuable contribution to the field. It highlights the underestimation of TBE cases in Poland and underscores the need for improved diagnostic criteria and follow-up testing to ensure accurate reporting. The study's findings should prompt public health officials to enhance surveillance and education efforts about TBE, particularly in regions previously considered low-risk.

Overall, the paper is a significant step forward in understanding and addressing TBE underreporting in Poland, and with the suggested improvements, it can provide even more robust data for public health interventions.

**********

6. PLOS authors have the option to publish the peer review history of their article (what does this mean? ). If published, this will include your full peer review and any attached files.

If you choose “no”, your identity will remain anonymous but your review may still be made public.

**Do you want your identity to be public for this peer review?** For information about this choice, including consent withdrawal, please see our Privacy Policy .

Reviewer #1: No

Reviewer #2: No

Reviewer #3: No

**********

[NOTE: If reviewer comments were submitted as an attachment file, they will be attached to this email and accessible via the submission site. Please log into your account, locate the manuscript record, and check for the action link "View Attachments". If this link does not appear, there are no attachment files.]

While revising your submission, please upload your figure files to the Preflight Analysis and Conversion Engine (PACE) digital diagnostic tool, https://pacev2.apexcovantage.com/ . PACE helps ensure that figures meet PLOS requirements. To use PACE, you must first register as a user. Registration is free. Then, login and navigate to the UPLOAD tab, where you will find detailed instructions on how to use the tool. If you encounter any issues or have any questions when using PACE, please email PLOS at figures@plos.org . Please note that Supporting Information files do not need this step.

# Author comment: Author response to Decision Letter 1

**DOI:** 10.1371/journal.pone.0323022.r003

8 Oct 2024

Reviewer 1

1. In my opinion, the manuscript presents a significant criticality: cases with positive cerebrospinal fluid serology cannot be considered on the same level as those with mere detection of serum antibodies. Furthermore, regarding the positivity in serum for IgM, it is worth noting the a sub-optimal specificity if compared to IgG performance. Before considering these IgM cases as true positives, the authors should have at least observed seroconversion.

Thank you for highlighting these important aspects of our paper. We agree that the serological methods employed are not entirely sufficient for the clinical diagnosis of TBE. However, our primary aim was to conduct an epidemiological study, not to develop new diagnostic methods for TBE. All of the patients included in our study exhibited symptoms consistent with neuroinfection. It is also important to emphasize that TBEV is the only flavivirus endemic to Poland. According to Prof. Gerhard Dobler (Chapter "Diagnostics of TBEV Infections" in the 7th edition of The TBE Book), ELISA tests demonstrate high sensitivity (up to 99%), but only moderate specificity due to cross-reactivity with other flaviviruses. Nevertheless, specificity can reach up to 97% when testing samples from individuals with no prior exposure to other flaviviruses, as in the case of our research. Furthermore, the comparison of specificity between IgM and IgG tests remains unclear. In the chapter mentioned above, Prof. Dobler notes: “Diagnostic tests for anti-TBEV IgM are usually more specific than IgG tests with regard to cross-reactivity with other flaviviruses (the author’s personal observation).”

It would indeed be valuable to analyze seroconversion over time; however, our study involved many small hospitals. Although this provided a comprehensive view of TBE epidemiology in areas outside of major cities—where many TBE infections occur—it also posed certain limitations. Collaborating with non-academic hospitals required us to adopt simplified diagnostic criteria that all participating institutions could follow, which unfortunately prevented us from observing seroconversion in all patients.

We described the suggested limitations in our Limitations section:

“Although all of our patients exhibited symptoms consistent with neuroinfection, in some cases, the diagnosis of TBE was based solely on elevated IgM or IgG levels. This alone is insufficient for a definitive diagnosis [18]. However, since TBEV is the only flavivirus endemic to Poland, and we excluded patients vaccinated against flaviviruses while also collecting data on any potential travel-related exposure, we believe that, under these conditions, the specificity (>95%) and sensitivity (>95%) of the ELISA test are adequate for epidemiological purposes [23].”

We have also added more description of our diagnostic criteria:

“All our patients diagnosed with TBE exhibited symptoms of neuroinfection and met at least one laboratory criterion, defined as the presence of IgM or IgG antibodies in blood or cerebrospinal fluid. These criteria differ from the official TBE case confirmation guidelines, which require clinical symptoms alongside both positive IgM and IgG in the blood, or IgM in the cerebrospinal fluid [18]. In our study, we classified patients with only IgM or IgG detected in the blood as TBE cases, as our goal was to conduct an epidemiological study that included as many local hospitals as possible. Many of these hospitals do not perform extended TBE diagnostic procedures, such as waiting for IgG seroconversion, particularly given the lack of specific treatment for TBE [19]. To minimize the risk of false-positive diagnoses, we excluded all patients with other potential causes of elevated IgM, such as autoimmune diseases, neoplasms, or prior vaccination against Flaviviridae viruses. Additionally, it is important to note that TBEV is the only flavivirus present in Poland.” 

###################

Reviewer 2

1. Results: Please mention that the 124 patients with TBEV antibodies were not previously vaccinated against TBEV.

Thank you for this important suggestion. We have underlined it:

“Throughout the study period, from April 1, 2018, to December 31, 2022, we included 766 patients in our analysis, of whom 124 were diagnosed with TBE. None of them were vaccinated against TBE.”

And also in Abstract:

“Among 766 patients, 124 exhibited serum or CSF antibodies against TBEV. None of them were vaccinated against TBE.”

2. Introduction: The family name Flaviviridae should be in italics.

We have implemented your suggestion.

3. Mortality Rate: The mortality rate of 1-5% is often mentioned in the literature, but nowadays it is usually less than 1% in most European countries. Please update this information.

Thank you for the suggestion. We have clarified that, for the European strain of TBEV, the mortality rate is less than 1%:

“Although some cases of TBE may remain asymptomatic, a significant proportion, ranging from 35% to 58%, can lead to the development of long-term neurological sequelae, with a mortality of European TBEV lower than 1% [3]”

4. Methods: It is not clear why only patients who received the TBEV vaccine in the last six months were excluded. I would expect that all TBEV vaccinated individuals would be excluded. Please explain.

Thank you for bringing this to our attention. We agree that prior vaccination is a significant factor in the context of our study. After reevaluating the data, we confirmed that no previously vaccinated patients were included, even those vaccinated more than six months before hospital admission. We have made the following changes to the Methods section:

“Patients who had received a flavivirus vaccination or did not provide their consent to participate were excluded from the study.”

We would also like to emphasize that, although Poland is an endemic region for TBE, only 1–2% of the population is vaccinated against the virus.

5. Laboratory Analysis: Please specify the kits used for IgG and IgM, including the borderline and positivity values. Also, it would be good to mention the case definition (for example, according to Taba et al., Eur J Neurol. 2017 Oct;24(10):1214-e61. doi: 10.1111/ene.13356.) and then mention in the results/discussion if the newly identified patients indeed meet the case definition.

Thank you for the suggestion. We have added a paragraph to the discussion addressing our diagnostic criteria. Although these criteria were not sufficient for a clinical diagnosis of TBE, our primary aim was to conduct an epidemiological study:

“All our patients diagnosed with TBE exhibited symptoms of neuroinfection and met at least one laboratory criterion, defined as the presence of IgM or IgG antibodies in blood or cerebrospinal fluid. These criteria differ from the official TBE case confirmation guidelines, which require clinical symptoms alongside both positive IgM and IgG in the blood, or IgM in the cerebrospinal fluid [18]. In our study, we classified patients with only IgM or IgG detected in the blood as TBE cases, as our goal was to conduct an epidemiological study that included as many local hospitals as possible. Many of these hospitals do not perform extended TBE diagnostic procedures, such as waiting for IgG seroconversion, particularly given the lack of specific treatment for TBE [19]. To minimize the risk of false-positive diagnoses, we excluded all patients with other potential causes of elevated IgM, such as autoimmune diseases, neoplasms, or prior vaccination against Flaviviridae viruses. Additionally, it is important to note that TBEV is the only flavivirus present in Poland.”

Additionally, we discussed it in our limitation section:

“Although all of our patients exhibited symptoms consistent with neuroinfection, in some cases, the diagnosis of TBE was based solely on elevated IgM or IgG levels. This alone is insufficient for a definitive diagnosis [18]. However, since TBEV is the only flavivirus endemic to Poland, and we excluded patients vaccinated against flaviviruses while also collecting data on any potential travel-related exposure, we believe that, under these conditions, the specificity (>95%) and sensitivity (>95%) of the ELISA test are adequate for epidemiological purposes [23].”

We have also extended the description of serological diagnosis:

“Serological tests were performed on serum or CSF samples. Patients with clinical symptoms of neuroinfection and a positive result for either IgM or IgG in serum, or IgM or IgG in cerebrospinal fluid (CSF) were diagnosed as TBE patients. To identify anti-TBE immunoglobulins, we used the enzyme-linked immunosorbent assay (ELISA) method, using Virotech ELISA kits (Germany). All analyses were performed at the Immunoserology Laboratory of the Medical University of Białystok. At the same time, patients were required to complete a questionnaire that captured clinical and epidemiological data.”

6. Clinical Characteristics: It is mentioned here that none of the diagnosed patients had received vaccination against TBEV, but in the methods, it is mentioned that only patients who received the vaccine within the last six months were excluded. Please clarify this discrepancy.

Thank you for identifying this discrepancy. We have clarified the issue as outlined in your previous question.

7. Discussion: It would be beneficial to briefly mention the situation in neighboring countries, such as Germany, the Czech Republic, and Slovakia, if this information is available from the literature.

Thank you for this interesting suggestion. We have included additional information in the discussion and added a reference to the ECDC report:

“Our results show that the epidemiological situation in Poland does not differ much from that in Lithuania, Slovakia, the Czech Republic, or Austria, where, according to the European Centre for Disease Prevention and Control (ECDC), the entire countries are considered at risk for TBE infections, with a risk level similar to that of endemic regions in Poland.”

###################

Reviewer 3

1. The authors state that they detected IgM antibodies in serum or CSF samples to confirm TBE infection. However, they do not specify the criteria or definition they followed to confirm TBE as the cause of neuroinfection. This lack of specification raises questions about the accuracy of the diagnosis.

The presence of IgM antibodies alone is not a definitive confirmation of TBE. IgM antibodies can yield false-positive results, especially in patients with other health conditions such as autoimmune diseases or mycoplasma infections, which the paper acknowledges were present in some patients. To address this, it would be prudent to follow up with IgM-positive patients to check for seroconversion to IgG antibodies. This follow-up would help rule out false positives and provide a more accurate assessment of TBE incidence.

Thank you for this important suggestion. We have added the case definition to our Methods section:

“Patients with clinical symptoms of neuroinfection and a positive result for either IgM or IgG in serum, or IgM or IgG in cerebrospinal fluid (CSF) were diagnosed as TBE patients.”

We have also discussed the difference between our case definition and official recommendations:

“All our patients diagnosed with TBE exhibited symptoms of neuroinfection and met at least one laboratory criterion, defined as the presence of IgM or IgG antibodies in blood or cerebrospinal fluid. These criteria differ from the official TBE case confirmation guidelines, which require clinical symptoms alongside both positive IgM and IgG in the blood, or IgM in the cerebrospinal fluid [18]. In our study, we classified patients with only IgM or IgG detected in the blood as TBE cases, as our goal was to conduct an epidemiological study that included as many local hospitals as possible. Many of these hospitals do not perform extended TBE diagnostic procedures, such as waiting for IgG seroconversion, particularly given the lack of specific treatment for TBE [19]. To minimize the risk of false-positive diagnoses, we excluded all patients with other potential causes of elevated IgM, such as autoimmune diseases, neoplasms, or prior vaccination against Flaviviridae viruses. Additionally, it is important to note that TBEV is the only flavivirus present in Poland.” 

We have also discussed the limitations of our study caused by this case definition:

“Although all of our patients exhibited symptoms consistent with neuroinfection, in some cases, the diagnosis of TBE was based solely on elevated IgM or IgG levels. This alone is insufficient for a definitive diagnosis [18]. However, since TBEV is the only flavivirus endemic to Poland, and we excluded patients vaccinated against flaviviruses while also collecting data on any potential travel-related exposure, we believe that, under these conditions, the specificity (>95%) and sensitivity (>95%) of the ELISA test are adequate for epidemiological purposes [23].”

Attachment Submitted filename: TBE_review_response_ver2.docx

# Peer review: Decision Letter 1

**DOI:** 10.1371/journal.pone.0323022.r004

**Authors:** Oluyinka Ajibola Iyiola

21 Nov 2024

PONE-D-24-01666R1The Impact of Serological Testing Implementation on Tick-Borne Encephalitis Detection in PolandPLOS ONE

Dear Dr. Wawrzuta,

Thank you for submitting your manuscript to PLOS ONE. After careful consideration, we feel that it has merit but does not fully meet PLOS ONE’s publication criteria as it currently stands. Therefore, we invite you to submit a revised version of the manuscript that addresses the points raised during the review process.

Please submit your revised manuscript by Jan 05 2025 11:59PM. If you will need more time than this to complete your revisions, please reply to this message or contact the journal office at plosone@plos.org . When you're ready to submit your revision, log on to https://www.editorialmanager.com/pone/ and select the 'Submissions Needing Revision' folder to locate your manuscript file.

Please include the following items when submitting your revised manuscript:

- A rebuttal letter that responds to each point raised by the academic editor and reviewer(s). You should upload this letter as a separate file labeled 'Response to Reviewers'.
- A marked-up copy of your manuscript that highlights changes made to the original version. You should upload this as a separate file labeled 'Revised Manuscript with Track Changes'.
- An unmarked version of your revised paper without tracked changes. You should upload this as a separate file labeled 'Manuscript'.

If you would like to make changes to your financial disclosure, please include your updated statement in your cover letter. Guidelines for resubmitting your figure files are available below the reviewer comments at the end of this letter.

If applicable, we recommend that you deposit your laboratory protocols in protocols.io to enhance the reproducibility of your results. Protocols.io assigns your protocol its own identifier (DOI) so that it can be cited independently in the future. For instructions, see: https://journals.plos.org/plosone/s/submission-guidelines#loc-laboratory-protocols . Additionally, PLOS ONE offers an option for publishing peer-reviewed Lab Protocol articles, which describe protocols hosted on protocols.io. Read more information on sharing protocols at https://plos.org/protocols?utm_medium=editorial-email&utm_source=authorletters&utm_campaign=protocols .

We look forward to receiving your revised manuscript.

Kind regards,

Dr. Oluyinka Ajibola IYIOLA

Academic Editor

PLOS ONE

Journal Requirements:

Please review your reference list to ensure that it is complete and correct. If you have cited papers that have been retracted, please include the rationale for doing so in the manuscript text, or remove these references and replace them with relevant current references. Any changes to the reference list should be mentioned in the rebuttal letter that accompanies your revised manuscript. If you need to cite a retracted article, indicate the article’s retracted status in the References list and also include a citation and full reference for the retraction notice.

[Note: HTML markup is below.]. Please do not edit.

Reviewers' comments:

Reviewer's Responses to Questions

**Comments to the Author**

1. If the authors have adequately addressed your comments raised in a previous round of review and you feel that this manuscript is now acceptable for publication, you may indicate that here to bypass the “Comments to the Author” section, enter your conflict of interest statement in the “Confidential to Editor” section, and submit your "Accept" recommendation.

Reviewer #4: All comments have been addressed

**********

2. Is the manuscript technically sound, and do the data support the conclusions?

The manuscript must describe a technically sound piece of scientific research with data that supports the conclusions. Experiments must have been conducted rigorously, with appropriate controls, replication, and sample sizes. The conclusions must be drawn appropriately based on the data presented. 

Reviewer #4: Yes

**********

3. Has the statistical analysis been performed appropriately and rigorously? 

Reviewer #4: Yes

**********

4. Have the authors made all data underlying the findings in their manuscript fully available?

The PLOS Data policy requires authors to make all data underlying the findings described in their manuscript fully available without restriction, with rare exception (please refer to the Data Availability Statement in the manuscript PDF file). The data should be provided as part of the manuscript or its supporting information, or deposited to a public repository. For example, in addition to summary statistics, the data points behind means, medians and variance measures should be available. If there are restrictions on publicly sharing data—e.g. participant privacy or use of data from a third party—those must be specified.

Reviewer #4: Yes

**********

5. Is the manuscript presented in an intelligible fashion and written in standard English?

PLOS ONE does not copyedit accepted manuscripts, so the language in submitted articles must be clear, correct, and unambiguous. Any typographical or grammatical errors should be corrected at revision, so please note any specific errors here.

Reviewer #4: Yes

**********

6. Review Comments to the Author

Please use the space provided to explain your answers to the questions above. You may also include additional comments for the author, including concerns about dual publication, research ethics, or publication ethics. Please upload your review as an attachment if it exceeds 20,000 characters.

Reviewer #4: There are defect of key areas. The Factors of socio-demography: what role does it play in the prevalence or incidence of TBE in the region? What were the findings of previous study and how did that influence or affect the current study. Is there a correlation in result or is this a fresh gap that has been identified?

The introduction fails to establish the demography of the target audience as well as the epidemiology .How does other studies done influence the study at hand. Were there critical literature reviews and

How does it affect the current finding?

What other literature shows the gap?

While discussing the Objective of the study, what is the relationship between the Independent and dependent variables. How does the independent variable affect the outcome of the study?

The study fails to elucidate the reason behind the choice of the Methodology used .What is the justification for the study location? How did you arrive at the study site. Why chose 29 departments and not 20? Was there a set of criteria that were used? What is the specific demography of the study site that warranted the choice?

While analyzing the discussion and the significant finding, it was not clear if it correlated with the Significant finding in

Literature Review.

The study has not defined the limit of study limitation .

The study has been able to reveal that there is an underreporting of incidence of TBE in non-endemic regions, and the awareness is low, leading to increased incidence and undertreatment of the diseases. It has advocated for strategies to stimulate increase in the knowledge and awareness of the diseases

**********

7. PLOS authors have the option to publish the peer review history of their article (what does this mean? ). If published, this will include your full peer review and any attached files.

If you choose “no”, your identity will remain anonymous, but your review may still be made public.

**Do you want your identity to be public for this peer review?** For information about this choice, including consent withdrawal, please see our Privacy Policy .

Reviewer #4: **Yes: ** Adekunle Megbuwawon

**********

[NOTE: If reviewer comments were submitted as an attachment file, they will be attached to this email and accessible via the submission site. Please log into your account, locate the manuscript record, and check for the action link "View Attachments". If this link does not appear, there are no attachment files.]

While revising your submission, please upload your figure files to the Preflight Analysis and Conversion Engine (PACE) digital diagnostic tool, https://pacev2.apexcovantage.com/ . PACE helps ensure that figures meet PLOS requirements. To use PACE, you must first register as a user. Registration is free. Then, login and navigate to the UPLOAD tab, where you will find detailed instructions on how to use the tool. If you encounter any issues or have any questions when using PACE, please email PLOS at figures@plos.org . Please note that Supporting Information files do not need this step.

# Author comment: Author response to Decision Letter 2

**DOI:** 10.1371/journal.pone.0323022.r005

6 Dec 2024

Thank you for your thorough review and valuable suggestions. We have addressed each of your comments in detail below.

1. The Factors of socio-demography: what role does it play in the prevalence or incidence of TBE in the region? What were the findings of previous study and how did that influence or affect the current study. Is there a correlation in result or is this a fresh gap that has been identified?

Thank you for raising this important question. In the literature, TBE risk is generally not associated with socio-demographic factors, as the primary determinants of risk are environmental conditions and activities that increase tick exposure. According to the most recent Annual Epidemiological Report from the European Centre for Disease Prevention and Control (ECDC), TBE cases are more frequently reported among men (male-to-female ratio: 1.5:1), with the highest incidence observed in the 45–64 age group (https://www.ecdc.europa.eu/en/publications-data/tick-borne-encephalitis-annual-epidemiological-report-2022)

Previous studies indicate that these demographic differences are likely driven by patterns of occupational and recreational exposure. Specifically, spending over 10 hours per week in forests—either for work or leisure—is a known risk factor for TBE in endemic regions (10.1371/journal.pone.0045511)

We have incorporated this context into the Introduction section of our manuscript as follows:

“In Europe, TBE cases are more frequently reported among men, particularly in the 45–64 age group. This pattern is likely explained by increased exposure to forested environments during work or recreational activities, as spending more than 10 hours per week in forests within endemic regions is a recognized risk factor for TBE infection.”

2. The introduction fails to establish the demography of the target audience as well as the epidemiology .How does other studies done influence the study at hand. Were there critical literature reviews and how does it affect the current finding?

What other literature shows the gap?

Thank you for this important comment. We have added a new paragraph to the Introduction, which describes the demographic aspects and integrates previous theoretical research that serves as the foundation for our empirical study.

“Currently, two regions in Poland—Podlaskie and Warmińsko-Mazurskie voivodeships—are recognized as endemic for TBE. These regions are home to over 2.5 million people, with approximately 30% of the land area covered by forests. As popular tourist destinations, these areas attract around 1.5 million visitors annually, further increasing potential exposure to ticks. However, a theoretical analysis conducted by Stefanoff et al. suggested that the entire territory of Poland could be considered endemic for TBE. To date, these theoretical findings have not been empirically confirmed.”

3. While discussing the Objective of the study, what is the relationship between the Independent and dependent variables. How does the independent variable affect the outcome of the study?

Thank you for this thoughtful observation. In our study, we do not operate with traditional independent and dependent variables, as our primary objective is to estimate the proportion of neuroinfections of unknown origin that are, in fact, TBE infections. This approach is intended to provide a more accurate representation of the true TBE incidence in Poland. To emphasize this, we have revised our "Aim of the Study" as follows:

“This study aims to determine the true prevalence of TBE in traditionally non-endemic regions of Poland by conducting systematic serological analyses of serum and cerebrospinal fluid samples obtained from patients presenting with unexplained neuroinfections. The ultimate goals are to identify emerging TBE hotspots and assess potential underreporting by comparing the findings with official statistics.” 

4. The study fails to elucidate the reason behind the choice of the Methodology used. What is the justification for the study location? How did you arrive at the study site. Why chose 29 departments and not 20? Was there a set of criteria that were used? What is the specific demography of the study site that warranted the choice?

Thank you for raising this point. We have expanded the description in the Methods section to address all your questions:

“We invited all departments treating neuroinfections from regions identified as potentially having a higher-than-reported TBE incidence to participate in the study. Ultimately, 29 departments agreed to take part, including infectious disease units and neurology departments specializing in diagnosing and treating neuroinfections. These centers were strategically located in areas with limited official reporting of TBE cases, but preliminary data indicated the potential presence of undiagnosed TBE cases. Furthermore, these departments had the capacity to perform the serological tests required for this study. The Department of Infectious Diseases and Neuroinfections at the Medical University of Białystok oversaw and coordinated the participation of all included departments."

5. While analyzing the discussion and the significant finding, it was not clear if it correlated with the Significant finding in Literature Review.

The study has not defined the limit of study limitation .

Thank you for this valuable comment. We have revised the Discussion section to better contextualize our findings within the theoretical framework proposed by Stefanoff et al. (10.1371/journal.pone.0045511) and to address the study limitations.

“Stefanoff et al. [10] pioneered the modeling of TBE risk in Poland and created a TBE risk map that identified potential new risk areas with no officially reported TBE cases. Our findings align with and build upon this foundational work, providing evidence that challenges the previous assumption that only a few regions in Poland are endemic. Specifically, our study identified that 16% of patients presenting with unexplained central nervous system infections in regions previously considered non-endemic were, in fact, diagnosed with TBE. These results support Stefanoff et al.’s assertion that more regions in Poland should be classified as TBE endemic.”

We have also extended one paragraph in Limitations

“Several limitations are inherent to our study. The relatively low sample count from certain voivodeships could have influenced the proportional representation of positive samples in those regions, potentially leading to a misstatement of TBE prevalence. Furthermore, the impact of the COVID-19 pandemic on healthcare systems, including the activity of infectious diseases departments, introduced an external factor that could have affected patient inclusion rates and testing patterns. The absence of comprehensive epidemiological investigations in our study precluded a thorough understanding of the precise sources of infection among patients, leaving room for potential biases in exposure assessments. Our study is also constrained by its focus on specific geographic areas, which may not capture the full extent of underdiagnosed TBE cases.”

Attachment Submitted filename: review2_response.docx

# Peer review: Decision Letter 2

**DOI:** 10.1371/journal.pone.0323022.r006

**Authors:** Oluyinka Ajibola Iyiola

6 Feb 2025

PONE-D-24-01666R2The Impact of Serological Testing Implementation on Tick-Borne Encephalitis Detection in PolandPLOS ONE

Dear Dr. Wawrzuta,

Thank you for submitting your manuscript to PLOS ONE. After careful consideration, we feel that it has merit but does not fully meet PLOS ONE’s publication criteria as it currently stands. Therefore, we invite you to submit a revised version of the manuscript that addresses the points raised during the review process.

Please submit your revised manuscript by Mar 23 2025 11:59PM. If you will need more time than this to complete your revisions, please reply to this message or contact the journal office at plosone@plos.org . When you're ready to submit your revision, log on to https://www.editorialmanager.com/pone/ and select the 'Submissions Needing Revision' folder to locate your manuscript file.

Please include the following items when submitting your revised manuscript:

- A rebuttal letter that responds to each point raised by the academic editor and reviewer(s). You should upload this letter as a separate file labeled 'Response to Reviewers'.
- A marked-up copy of your manuscript that highlights changes made to the original version. You should upload this as a separate file labeled 'Revised Manuscript with Track Changes'.
- An unmarked version of your revised paper without tracked changes. You should upload this as a separate file labeled 'Manuscript'.

If you would like to make changes to your financial disclosure, please include your updated statement in your cover letter. Guidelines for resubmitting your figure files are available below the reviewer comments at the end of this letter.

If applicable, we recommend that you deposit your laboratory protocols in protocols.io to enhance the reproducibility of your results. Protocols.io assigns your protocol its own identifier (DOI) so that it can be cited independently in the future. For instructions see: https://journals.plos.org/plosone/s/submission-guidelines#loc-laboratory-protocols . Additionally, PLOS ONE offers an option for publishing peer-reviewed Lab Protocol articles, which describe protocols hosted on protocols.io. Read more information on sharing protocols at https://plos.org/protocols?utm_medium=editorial-email&utm_source=authorletters&utm_campaign=protocols .

We look forward to receiving your revised manuscript.

Kind regards,

Oluyinka Ajibola Iyiola

Academic Editor

PLOS ONE

Journal Requirements:

Please review your reference list to ensure that it is complete and correct. If you have cited papers that have been retracted, please include the rationale for doing so in the manuscript text, or remove these references and replace them with relevant current references. Any changes to the reference list should be mentioned in the rebuttal letter that accompanies your revised manuscript. If you need to cite a retracted article, indicate the article’s retracted status in the References list and also include a citation and full reference for the retraction notice.

[Note: HTML markup is below. Please do not edit.]

Reviewers' comments:

Reviewer's Responses to Questions

**Comments to the Author**

1. If the authors have adequately addressed your comments raised in a previous round of review and you feel that this manuscript is now acceptable for publication, you may indicate that here to bypass the “Comments to the Author” section, enter your conflict of interest statement in the “Confidential to Editor” section, and submit your "Accept" recommendation.

Reviewer #2: All comments have been addressed

Reviewer #3: All comments have been addressed

Reviewer #5: All comments have been addressed

Reviewer #6: (No Response)

**********

2. Is the manuscript technically sound, and do the data support the conclusions?

The manuscript must describe a technically sound piece of scientific research with data that supports the conclusions. Experiments must have been conducted rigorously, with appropriate controls, replication, and sample sizes. The conclusions must be drawn appropriately based on the data presented. 

Reviewer #2: Yes

Reviewer #3: Yes

Reviewer #5: Yes

Reviewer #6: No

**********

3. Has the statistical analysis been performed appropriately and rigorously? 

Reviewer #2: N/A

Reviewer #3: Yes

Reviewer #5: I Don't Know

Reviewer #6: No

**********

4. Have the authors made all data underlying the findings in their manuscript fully available?

The PLOS Data policy requires authors to make all data underlying the findings described in their manuscript fully available without restriction, with rare exception (please refer to the Data Availability Statement in the manuscript PDF file). The data should be provided as part of the manuscript or its supporting information, or deposited to a public repository. For example, in addition to summary statistics, the data points behind means, medians and variance measures should be available. If there are restrictions on publicly sharing data—e.g. participant privacy or use of data from a third party—those must be specified.

Reviewer #2: Yes

Reviewer #3: Yes

Reviewer #5: No

Reviewer #6: No

**********

5. Is the manuscript presented in an intelligible fashion and written in standard English?

PLOS ONE does not copyedit accepted manuscripts, so the language in submitted articles must be clear, correct, and unambiguous. Any typographical or grammatical errors should be corrected at revision, so please note any specific errors here.

Reviewer #2: Yes

Reviewer #3: Yes

Reviewer #5: Yes

Reviewer #6: Yes

**********

6. Review Comments to the Author

Please use the space provided to explain your answers to the questions above. You may also include additional comments for the author, including concerns about dual publication, research ethics, or publication ethics. (Please upload your review as an attachment if it exceeds 20,000 characters)

Reviewer #2: The authors addressed all my comments. I have no further comments to consider. I recommend accepting the manuscript in the present form.

Reviewer #3: The authors have addressed most of my concerns satisfactorily, particularly in terms of study limitations. However, the issue of relying on IgM positivity without IgG seroconversion remains a gap, what leaves room for potential diagnostic inaccuracies.

Reviewer #5: Dear authors,

This paper provides important information on the distribution and incidence of tick-borne encephalitis virus in Poland. The research gives insights into the epidemiology and geographic spread of tick-borne encephalitis virus in Poland, which is relevant to a public health approach.

Here are my comments on the manuscript:

Paragraph three introduction: This new text does not fit well into the introduction. Please consider rewriting it.

Aim of the study: In relation to this new text, please consider revising or removing the term "true prevalence," as it may not be appropriate given the study design and its limitations.

Methods/included centers: Could you provide information about the centers or areas not covered in your study? Are there any data limitations related to this point?

Laboratory analysis: Why was a TBEV neutralization test not included to confirm the positive samples? Obtaining results from this test would have provided valuable insights into the level of neutralizing antibodies in the patients. However, I recognize that there may have been limitations, such as limited sample volume, which prevented this analysis.

Discussion:

Paragraph 3:Include a reference to the specific ELISA test used in this study along with reference 27. The specificity and sensitivity of the test should take into account its cross-reactivity with related flaviviruses.

Limitations and future research: Please include information regarding cross-reactions in the ELISA test for other flaviviruses. How can it be confirmed that this is caused by TBEV? It could potentially be other unknown flaviviruses. Additionally, provide more details about related or possible travel-related cases, particularly for patients who are only IgG positive.

Reviewer #6: Dear authors,

while I think that the data presented is important and adresses a crucial topic for public health, I also think that the data presented here is not transperently enough presented in its current form.

**********

7. PLOS authors have the option to publish the peer review history of their article (what does this mean? ). If published, this will include your full peer review and any attached files.

If you choose “no”, your identity will remain anonymous but your review may still be made public.

**Do you want your identity to be public for this peer review?** For information about this choice, including consent withdrawal, please see our Privacy Policy .

Reviewer #2: No

Reviewer #3: No

Reviewer #5: No

Reviewer #6: No

**********

[NOTE: If reviewer comments were submitted as an attachment file, they will be attached to this email and accessible via the submission site. Please log into your account, locate the manuscript record, and check for the action link "View Attachments". If this link does not appear, there are no attachment files.]

While revising your submission, please upload your figure files to the Preflight Analysis and Conversion Engine (PACE) digital diagnostic tool, https://pacev2.apexcovantage.com/ . PACE helps ensure that figures meet PLOS requirements. To use PACE, you must first register as a user. Registration is free. Then, login and navigate to the UPLOAD tab, where you will find detailed instructions on how to use the tool. If you encounter any issues or have any questions when using PACE, please email PLOS at figures@plos.org . Please note that Supporting Information files do not need this step.

Attachment Submitted filename: Review PLOS.docx

# Author comment: Author response to Decision Letter 3

**DOI:** 10.1371/journal.pone.0323022.r007

9 Feb 2025

Reviewer 2

Thank you very much for your thorough review and constructive feedback. We appreciate your detailed comments.

################## 

Reviewer 3

Thank you very much for your comprehensive suggestions and thoughtful insights. We believe that implementing these changes has significantly improved our manuscript's clarity and scientific rigor.

1. “However, the issue of relying on IgM positivity without IgG seroconversion remains a gap, what leaves room for potential diagnostic inaccuracies.”

Thank you for raising this important point about IgM/IgG diagnostics. We have carefully considered the potential limitations of relying on IgM positivity and have taken several steps to ensure diagnostic accuracy. As we discuss in the manuscript, TBEV is the only Flavivirus in Poland that causes CNS infections. However, in response to your valid concern, we have expanded this section of the Discussion and added key references documenting the absence of other Flaviviridae infections in Poland:

“To minimize the risk of false-positive diagnoses, we excluded all patients with other potential causes of elevated IgM, such as autoimmune diseases, neoplasms, or prior vaccination against Flaviviridae viruses. Furthermore, TBE virus is currently the only endemic flavivirus in Poland, as epidemiological surveillance has not detected West Nile virus or other flaviviruses as causes of encephalitis cases in the country [24,25].”

################## 

Reviewer 5

Thank you for your thorough and insightful review. We appreciate your detailed suggestions for improving our manuscript. We have carefully considered each point and provided our responses below.

1.“This new text does not fit well into the introduction. Please consider rewriting it.”

Thank you for this helpful observation regarding the text flow. We have restructured the Introduction to improve its coherence by merging related content about therapies and vaccination into a single, cohesive paragraph and creating a separate, dedicated paragraph for epidemiological information.

2.“Aim of the study: In relation to this new text, please consider revising or removing the term "true prevalence," as it may not be appropriate given the study design and its limitations.”

Thank you for this suggestion. We have changed it as follows:

“This study aims to evaluate the occurrence and distribution of TBE in traditionally non-endemic regions of Poland by conducting systematic serological analyses of serum and cerebrospinal fluid samples obtained from patients presenting with unexplained neuroinfections. The ultimate goals are to identify emerging TBE hotspots and assess potential underreporting by comparing the findings with official statistics.”

3.“Methods/included centers: Could you provide information about the centers or areas not covered in your study? Are there any data limitations related to this point?”

Thank you for this important question about geographic coverage. We have elaborated on this limitation in our revised manuscript:

“Our study has geographic limitations, as it focused primarily on regions traditionally considered non-endemic for TBE. We intentionally excluded centers from known endemic areas to specifically evaluate TBE occurrence in presumed non-endemic regions. While this approach served our primary research objective, it means we cannot provide a comprehensive national picture of TBE incidence. It's possible that applying similar systematic testing protocols in endemic regions could reveal even higher incidence rates than currently reported, although healthcare providers in these areas typically maintain higher awareness and vigilance for TBE cases”

4.“Laboratory analysis: Why was a TBEV neutralization test not included to confirm the positive samples? Obtaining results from this test would have provided valuable insights into the level of neutralizing antibodies in the patients. However, I recognize that there may have been limitations, such as limited sample volume, which prevented this analysis.”

Thank you for this important point. We did not perform neutralization tests for several reasons:

1. Our primary goal was to conduct a large-scale epidemiological study involving multiple local hospitals. Adding neutralization testing would have significantly increased the complexity and cost of the study.

2. Given the absence of other endemic flaviviruses in Poland during the study period (as confirmed by national surveillance data showing no detection of West Nile virus or other flaviviruses), and our careful exclusion of patients with prior flavivirus vaccinations or potential cross-reactive conditions, we considered the specificity of ELISA testing sufficient for our epidemiological purposes.

3. The study's focus was on identifying potential TBE cases in non-endemic regions to demonstrate the need for enhanced surveillance, rather than conducting detailed serological characterization of each case.

5. “Discussion: Paragraph 3:Include a reference to the specific ELISA test used in this study along with reference 27. The specificity and sensitivity of the test should take into account its cross-reactivity with related flaviviruses. Limitations and future research: Please include information regarding cross-reactions in the ELISA test for other flaviviruses. How can it be confirmed that this is caused by TBEV? It could potentially be other unknown flaviviruses. Additionally, provide more details about related or possible travel-related cases, particularly for patients who are only IgG positive.”

Thank you for these important points about testing specificity and potential cross-reactivity. Our approach to ensuring accurate diagnosis was comprehensive. To minimize the risk of false-positive diagnoses, we excluded all patients with other potential causes of elevated IgM, such as autoimmune diseases, neoplasms, or prior vaccination against Flaviviridae viruses. Furthermore, TBE virus is currently the only endemic flavivirus in Poland, as epidemiological surveillance has not detected West Nile virus or other flaviviruses as causes of encephalitis cases in the country. We have added the description and references:

“To minimize the risk of false-positive diagnoses, we excluded all patients with other potential causes of elevated IgM, such as autoimmune diseases, neoplasms, or prior vaccination against Flaviviridae viruses. Furthermore, TBE virus is currently the only endemic flavivirus in Poland, as epidemiological surveillance has not detected West Nile virus or other flaviviruses as causes of encephalitis cases in the country [24,25].”

Regarding travel-related cases, we conducted detailed interviews with all patients about possible sources of infection. Only 3 of them traveled abroad – to Hungary, Corfu, and Croatia, as shown in Table 3. We have improved this description in the manuscript:

“Additionally, 11 patients were suspected of contracting the infection during travel (3 of them abroad – Hungary, Corfu or Croatia), and an additional five patients reported consuming unpasteurized dairy products. A comprehensive breakdown of these potential infection sources is provided in Table 3.”

################## 

Reviewer 6

Thank you very much for all your constructive comments. We have addressed each point and implemented the corresponding changes in the manuscript, as detailed below.

1.“Aims: What are “official statistics”?”

Regarding your question about 'official statistics', we have extended and clarified this explanation as follows:

“The ultimate goals are to identify emerging TBE hotspots and assess potential underreporting by comparing the findings with official statistics from the Polish National Institute of Public Health's mandatory surveillance system.”

2.“Methods: “How did you know that the regions are having “potentially higher then reported TBE incidence rates”?”

Thank you for this important point. We have clarified our methodology in the Methods section:

“We invited all departments treating neuroinfections from regions identified as potentially having a higher-than-reported TBE incidence to participate in the study. This selection was based on previous theoretical modeling studies by Stefanoff et al. [11], which identified potential high-risk areas through analysis of environmental factors conducive to TBEV transmission”

3.How many departments did you contact? Which ones were excluded and why did you not take the “risk areas” as a positive control?

Thank you for these important questions about study site selection. We contacted all departments treating neuroinfections in non-endemic regions of Poland. Out of these, 29 departments agreed to participate, as described in our Methods section:

“We invited all departments treating neuroinfections from regions identified as potentially having a higher-than-reported TBE incidence to participate in the study.”

Regarding the exclusion of 'risk areas' (endemic regions) as control groups, this decision was based on several methodological considerations:

- Our primary objective was to evaluate the occurrence of TBE in traditionally non-endemic regions where testing for TBEV is not routinely performed. In contrast, endemic regions already have established testing protocols and higher awareness among medical professionals.

- Including endemic regions as controls would not have served our main research goal, which was to assess potential underdiagnosis in areas where TBE is not typically suspected. In endemic regions, patients are already widely tested for this etiology.

- This was designed as an epidemiological study specifically focusing on detecting TBE cases among patients with unexplained neuroinfections in presumed non-endemic areas, rather than a comprehensive national survey of TBE incidence.

We have acknowledged this limitation in our manuscript:

“Our study has geographic limitations, as it focused primarily on regions traditionally considered non-endemic for TBE. We intentionally excluded centers from known endemic areas to specifically evaluate TBE occurrence in presumed non-endemic regions. While this approach served our primary research objective, it means we cannot provide a comprehensive national picture of TBE incidence. It's possible that applying similar systematic testing protocols in endemic regions could reveal even higher incidence rates than currently reported, although healthcare providers in these areas typically maintain higher awareness and vigilance for TBE cases”

4.“April 1st, December 31st

Thank you for this suggestion about date formatting. However, we have chosen to maintain the format 'April 1, 2018, and December 31, 2022' rather than using ordinal indicators (1st, 31st) because this format aligns with the widely accepted scientific writing conventions used in medical literature.

5.“Explain CFS in the first sentence of “Laboratory analysis” not later”

Thank you for finding this mistake. We have corrected it.

6.Can you give details of how Igg and IgM were measured? What was the cut off titre? Is it a commercial kit? Was the method unis ono in all laboratories?

Thank you for these important questions about our testing methodology. All serological analyses were conducted using a standardized approach. We used the commercially available Virotech ELISA kit (Germany) for detecting both IgG and IgM antibodies against TBEV, as described:

“To identify anti-TBE immunoglobulins, we used the enzyme-linked immunosorbent assay (ELISA) method, using Virotech ELISA kits (Germany).”

All analyses were centralized and performed at a single location - the Immunoserology Laboratory of the Medical University of Białystok to ensure consistency and eliminate inter-laboratory variability. This centralized testing approach eliminated potential technical discrepancies that could arise from multiple testing sites, as described:

“All analyses were performed at the Immunoserology Laboratory of the Medical University of Białystok.”

7.Were 124 included in the study, previously detected as tbe cases or did you detect that? Why did the clinicians not check for tbe themselves then?

Thank you for this important question about case identification. The 124 TBE cases were newly detected through our study, not previously diagnosed cases. Prior to our study, TBE testing was not routinely performed in regions considered non-endemic. This was due to the prevailing belief that TBE was limited to certain endemic regions in Poland, combined with the absence of specific antiviral treatment for TBE and limited awareness of TBE risk in these areas.

Our study was initiated based on theoretical modeling work by Stefanoff et al., which suggested that TBE risk might extend beyond traditionally recognized endemic regions. As we described in the paper, we included patients who “exhibited clinical indicators suggestive of TBE, such as inflammation of the central nervous system (manifesting as meningitis, meningoencephalitis, encephalomyelitis or encephaloradiculitis) but the specific type of neuroinfection was not confirmed. Initially, these patients were classified within epidemiological surveillance as cases of unspecified viral meningitis (coded A87, B00.3, B02.1), unspecified meningitis (coded G03), other viral-specific encephalitis (coded A83, A85, B00.4, B02.0, B25.8), or unspecified encephalitis (coded A86, G04.8, G04.9).”

8.Table 4 should be a map

Thank you for this suggestion, Figure 2 depicts data from Table 4.

9.The study would benefit from a statistical analysis. For someone not from Poland it is hard to understand which departments were selected, why and which departments are TBE hot spots. Geografical explanation would benefit the discussion to explain possible tbe movement. Further, can it be excluded in these patients that they did not travel abroad or were in other departmens before or even hot spot areas?

Thank you for these important points about geographical analysis and travel history. Our paper includes several visualization tools to help readers understand the geographical and epidemiological context of TBE in Poland. Figure 2 shows the voivodeships participating in our research and their respective percentages of positive cases, while Figures 3A-E present the official reported epidemiological situation across regions. These visualizations provide a clear picture of both our study's geographic scope and the broader epidemiological context.

We carefully documented potential exposure sources, including travel. As shown in Table 3, only 11 of our 124 detected TBE patients (less than 10%) reported travel history - 3 abroad and 8 to other regions of Poland. This means that for over 90% of cases, there was a clear connection between infection and place of residence. Regarding travel as a potential confounder, this remains epidemiologically significant even if some patients acquired TBE in other regions. The mobility of populations means that healthcare providers in all regions should maintain awareness of TBE during their diagnostic process, regardless of their location's endemic status.

10.Which also does not come out from this paper is what the authors consider a “risk area”, how many cases per habitants per year? Showing absence and presence of tbe does not define areas at risk.

Thank you for this important point about defining risk areas. We have added clarification in the Methods section that we used the World Health Organization's (WHO) standardized definition of TBE risk areas, which defines a risk area as having ≥5 cases/100,000 population/year (https://cdn.who.int/media/docs/default-source/immunization/position_paper_documents/tick-borne-encephalitis/who-pp-tbe-2011-summary.pdf?sfvrsn=de01b1fe_2)

11.Did the authors show only areas that were positive for tbe, were there also departments that are indeed without positive cases?

Thank you for this important question. All departments participating in our study identified at least one positive TBE case during the study period, as we have now clarified in the Results section: “What is important to notice, all included departments reported at least one positive case.” This finding is particularly significant for regions like Lubuskie voivodeship, which had no officially reported TBE cases prior to our study, as shown in

Attachment Submitted filename: PLOS_review3.docx

# Peer review: Decision Letter 3

**DOI:** 10.1371/journal.pone.0323022.r008

**Authors:** Babak Pakbin

1 Apr 2025

The Impact of Serological Testing Implementation on Tick-Borne Encephalitis Detection in Poland

PONE-D-24-01666R3

Dear Dr. Wawrzuta,

We’re pleased to inform you that your manuscript has been judged scientifically suitable for publication and will be formally accepted for publication once it meets all outstanding technical requirements.

Within one week, you’ll receive an e-mail detailing the required amendments. When these have been addressed, you’ll receive a formal acceptance letter and your manuscript will be scheduled for publication.

An invoice will be generated when your article is formally accepted. Please note, if your institution has a publishing partnership with PLOS and your article meets the relevant criteria, all or part of your publication costs will be covered. Please make sure your user information is up-to-date by logging into Editorial Manager at Editorial Manager®  and clicking the ‘Update My Information' link at the top of the page. If you have any questions relating to publication charges, please contact our Author Billing department directly at authorbilling@plos.org.

If your institution or institutions have a press office, please notify them about your upcoming paper to help maximize its impact. If they’ll be preparing press materials, please inform our press team as soon as possible -- no later than 48 hours after receiving the formal acceptance. Your manuscript will remain under strict press embargo until 2 pm Eastern Time on the date of publication. For more information, please contact onepress@plos.org.

Kind regards,

Babak Pakbin

Academic Editor

PLOS ONE

Additional Editor Comments (optional):

I have no more comments. 

Reviewers' comments:

Reviewer's Responses to Questions

**Comments to the Author**

1. If the authors have adequately addressed your comments raised in a previous round of review and you feel that this manuscript is now acceptable for publication, you may indicate that here to bypass the “Comments to the Author” section, enter your conflict of interest statement in the “Confidential to Editor” section, and submit your "Accept" recommendation.

Reviewer #2: All comments have been addressed

Reviewer #3: All comments have been addressed

**********

2. Is the manuscript technically sound, and do the data support the conclusions?

The manuscript must describe a technically sound piece of scientific research with data that supports the conclusions. Experiments must have been conducted rigorously, with appropriate controls, replication, and sample sizes. The conclusions must be drawn appropriately based on the data presented. 

Reviewer #2: Yes

Reviewer #3: Yes

**********

3. Has the statistical analysis been performed appropriately and rigorously? 

Reviewer #2: Yes

Reviewer #3: Yes

**********

4. Have the authors made all data underlying the findings in their manuscript fully available?

The PLOS Data policy requires authors to make all data underlying the findings described in their manuscript fully available without restriction, with rare exception (please refer to the Data Availability Statement in the manuscript PDF file). The data should be provided as part of the manuscript or its supporting information, or deposited to a public repository. For example, in addition to summary statistics, the data points behind means, medians and variance measures should be available. If there are restrictions on publicly sharing data—e.g. participant privacy or use of data from a third party—those must be specified.

Reviewer #2: Yes

Reviewer #3: Yes

**********

5. Is the manuscript presented in an intelligible fashion and written in standard English?

PLOS ONE does not copyedit accepted manuscripts, so the language in submitted articles must be clear, correct, and unambiguous. Any typographical or grammatical errors should be corrected at revision, so please note any specific errors here.

Reviewer #2: Yes

Reviewer #3: Yes

**********

6. Review Comments to the Author

Please use the space provided to explain your answers to the questions above. You may also include additional comments for the author, including concerns about dual publication, research ethics, or publication ethics. (Please upload your review as an attachment if it exceeds 20,000 characters)

Reviewer #2: As already indicated in my prevous report, the authors fully addressed all my comments and concerns and the manuscript is accepable for publication.

Reviewer #3: (No Response)

**********

7. PLOS authors have the option to publish the peer review history of their article (what does this mean? ). If published, this will include your full peer review and any attached files.

If you choose “no”, your identity will remain anonymous but your review may still be made public.

**Do you want your identity to be public for this peer review?** For information about this choice, including consent withdrawal, please see our Privacy Policy .

Reviewer #2: No

Reviewer #3: No

**********

# Editor report: Acceptance letter

**DOI:** 10.1371/journal.pone.0323022.r009

**Authors:** Babak Pakbin

PONE-D-24-01666R3

PLOS ONE

Dear Dr. Wawrzuta,

I'm pleased to inform you that your manuscript has been deemed suitable for publication in PLOS ONE. Congratulations! Your manuscript is now being handed over to our production team.

At this stage, our production department will prepare your paper for publication. This includes ensuring the following:

* All references, tables, and figures are properly cited

* All relevant supporting information is included in the manuscript submission,

* There are no issues that prevent the paper from being properly typeset

You will receive further instructions from the production team, including instructions on how to review your proof when it is ready. Please keep in mind that we are working through a large volume of accepted articles, so please give us a few days to review your paper and let you know the next and final steps.

Lastly, if your institution or institutions have a press office, please let them know about your upcoming paper now to help maximize its impact. If they'll be preparing press materials, please inform our press team within the next 48 hours. Your manuscript will remain under strict press embargo until 2 pm Eastern Time on the date of publication. For more information, please contact onepress@plos.org.

If we can help with anything else, please email us at customercare@plos.org.

Thank you for submitting your work to PLOS ONE and supporting open access.

Kind regards,

PLOS ONE Editorial Office Staff

on behalf of

Dr. PLOS Manuscript Reassignment

Staff Editor

PLOS ONE
